# A Methodology for the Rapid Qualification of Additively Manufactured Materials Based on Pore Defect Structures

**DOI:** 10.1007/s40192-024-00343-9

**Published:** 2024-02-27

**Authors:** Krzysztof S. Stopka, Andrew Desrosiers, Amber Andreaco, Michael D. Sangid

**Affiliations:** 1 School of Aeronautics and Astronautics, Purdue University, West Lafayette, IN 47907 USA; 2 GE Additive, Hamilton, OH 45011 USA

**Keywords:** Additive manufacturing, Porosity, Fatigue, Material qualification, Part certification, Characterization, Computational modeling, Non-destructive evaluation

## Abstract

Additive manufacturing (AM) can create net or near-net-shaped components while simultaneously building the material microstructure, therefore closely coupling forming the material and shaping the part in contrast to traditional manufacturing with distinction between the two processes. While there are well-heralded benefits to AM, the widespread adoption of AM in fatigue-limited applications is hindered by defects such as porosity resulting from off-nominal process conditions. The vast number of AM process parameters and conditions make it challenging to capture variability in porosity that drives fatigue design allowables during qualification. Furthermore, geometric features such as overhangs and thin walls influence local heat conductivity and thereby impact local defects and microstructure. Consequently, qualifying AM material within parts in terms of material properties is not always a straightforward task. This article presents an approach for rapid qualification of AM fatigue-limited parts and includes three main aspects: (1) seeding pore defects of specific size, distribution, and morphology into AM specimens, (2) combining non-destructive and destructive techniques for material characterization and mechanical fatigue testing, and (3) conducting microstructure-based simulations of fatigue behavior resulting from specific pore defect and microstructure combinations. The proposed approach enables simulated data to be generated to validate and/or augment experimental fatigue data sets with the intent to reduce the number of tests needed and promote a more rapid route to AM material qualification. Additionally, this work suggests a closer coupling between material qualification and part certification for determining material properties at distinct regions within an AM part.

## Introduction

The terms qualification and certification are often used when introducing new technologies into regulated fields, such as aerospace or medical [1–4]. Although the two terms are sometimes used interchangeably depending on the context, they have specific distinctions. Certification involves an external party evaluating and accepting a part design, providing written confirmation of its established level of repeatable capability. This certification process can be costly and time-consuming, depending on the complexity of the part or system-level design, making it desirable to minimize certification iterations. Material characteristics and behavior are integral to part design, particularly regarding thermal and mechanical properties. To determine these material properties, materials are qualified, often by the entity designing the part, through extensive material characterization, testing programs, and periodic surveillance testing to ensure repeatable material capability. Like certification efforts, qualification efforts can require significant resources, especially when attempting to qualify a new material and process simultaneously. Thus, any effort that may help reduce development and/or non-recurring engineering (NRE) costs associated with material qualification is of interest to various industries.

In traditional manufacturing, material qualification and part certification have historically been treated as two distinct activities. Conventional materials processing, such as casting and forging, often involves subtractive post-processing to achieve the final part dimensions. Material characterization and testing efforts during the material qualification stage are considered separate from part certification. In conventional methodology, the distribution of material properties is determined and used to establish the design allowables during the qualification stage, and these allowables are subsequently applied uniformly over the part during the certification stage. However, additive manufacturing (AM) has introduced a method to create material and, in some cases, final part design simultaneously, and thus, there is a growing interest in integrating material qualification and part certification efforts, particularly for critical, structural parts.

Fatigue is the most likely failure mechanism in the majority of structural applications [1, 2, 5]. As a material’s fatigue capability is driven by a combination of composition, microstructure, defect content, and loading conditions, assessing all potential variation that may exist in an AM part can be extremely onerous. This is amplified by the number of AM process parameters and conditions (e.g., powder state, laser power and speed, hatch spacing, layer thickness, part orientation and position on the build platform, process gas flow, machine used, etc.) that directly affect the material state and therefore influence the fatigue behavior. Fatigue simulations can be useful to test the various microstructure, defect, and loading conditions and complement the experimental testing campaign [6–8]. Therefore, this article presents a hybrid approach of experimental mechanical tests and microstructure-based simulations that may be employed to reduce reliance on traditional mechanical testing to assess fatigue as part of AM material qualification efforts. Byproducts of the AM build process, such as residual stresses [9, 10], rough as-built surfaces [11], and porosity [12–14], reduce the fatigue resistance of AM components and hinder their widespread implementation. The former two conditions may be alleviated with post-processing operations such as heat treatment and machining/polishing, respectively, but porosity is more challenging to remedy. Hot isostatic pressing (HIP) can reduce porosity but has difficulty eliminating pores with irregular morphology or near free surfaces [15–17]. The focus of this work is therefore on evaluating the detrimental effects of porosity on the fatigue resistance of metallic AM components. This integrated computational materials engineering (ICME) approach is demonstrated for laser powder bed fusion (L-PBF) processing of Ni-base alloy 718 (also known as IN718) due to its prevalence in the aerospace industry.

Previous AM qualification efforts have similarly leveraged simulations and experiments in an ICME-based approach. Thapliyal and Mishra [18] recently outlined a materials systems approach for AM qualification, specifically considering the multiscale hierarchy of material structure at both the micro and macro scale. They emphasized the need to understand the thermomechanical and kinetic aspects of AM and included alloy chemistry in the process-structure–property relationship [19]. Experimental characterization and computational modeling at multiple length scales was leveraged to optimize the post-processing heat treatment in alloy 718Plus as part of DARPA’s Open Manufacturing program [20, 21]. Mindt et al. [22] also optimized AM process parameters for this alloy to minimize porosity using multiscale and multiphysics simulations alongside experiments. Fatigue properties of alloy 718Plus were assessed using a Bayesian inference approach that leveraged experimental data and microstructure-sensitive simulations [23]. Megahed et al. [24] presented a comprehensive, rapid qualification ICME approach and determined AM process parameters to build a full scale rocket nozzle while achieving target porosity, mechanical strength, and geometric accuracy. Other ICME efforts toward qualification of AM examined the manufacturability of complex shapes [25, 26], the role of post-processing and solution annealing [27], and the integration of process, microstructure, and fatigue modeling in a single computational framework [28].

The guideline presented in this article leverages a combination of experiments and microstructure-based simulations intentionally seeded with porosity to expedite the qualification of AM components, and the structure of this work is outlined as follows. To provide context, the section “Fatigue-Limited Parts and Design Allowables” provides an explanation of fatigue design allowables, including their importance in qualification/certification as well as how they typically are created. Emphasizing the role of porosity in fatigue behavior, the section “Typical Porosity in Additive Manufacturing” discusses the various types of porosity in AM, their causes, and common methods for quantifying pores using non-destructive evaluation (NDE) techniques. The development and characterization of AM materials is addressed in the section “AM Material Development and Characterization”, with a focus on the concept of intentionally seeding pores that may not be detectable using production level NDE techniques. The microstructure-sensitive fatigue modeling framework, serving as the foundation for the hybrid experimental-model qualification approach, is detailed in the section “Fatigue Modeling.” The application of the hybrid methodology is demonstrated in sections “Application of the Hybrid Methodology to Fatigue Design Allowables” and “Application of the Hybrid Methodology for Zone-Based Life Analysis.” Finally, the “Conclusions” section provides a summary of the work.

## Fatigue-Limited Parts and Design Allowables

While fatigue failure can be caused by various factors, unintentional porosity is widely recognized as a primary contributor to premature material failures. This is no different for AM materials where porosity is a known defect type observed [12–14]. Understanding this impact is extremely important for applications, both from a business perspective, and more importantly, from a safety perspective. With regards to the business perspective, if a part was designed to last a specific number of cycles to meet service schedules that influence resource planning (e.g., spare parts, shop time, engineer/technician availability), early failures may result in unplanned maintenance cycles that impact schedule and revenue opportunities. Furthermore, if failure of the application can result in catastrophic failure of an overall system that may cause a risk to personal safety, having a clear and often conservative assessment of safe application lifespan is imperative to plan evaluations and replacement of hardware prior to reaching end of service life.

The phrase design allowable may be utilized in different contexts in scientific literature. For the sake of this discussion, the authors define design allowable as a nominally processed material’s bulk (i.e., no impact of surface features) fatigue capability via the use of a regression that represents a statistically relevant minimum expected life. In other words, designs must ensure operating conditions commensurate to the capability defined by the design allowable.

Creating such a design allowable requires significant empirical fatigue testing of material coupons, often trying to intentionally incorporate known sources of variation (e.g., compositional, orientation, production runs, etc.) that influence nominal test results. In such testing, unintentional sources of variation (e.g., porosity) may also be present; however, unintentional variation is not guaranteed to exist when processing all parts to nominal conditions. This has historically driven the requirement for test campaigns with large sample sizes (i.e., number of individual tests) to increase the probability of adequately capturing the unintentional variation for incorporation in the design allowable assessments.

Fatigue test campaigns are often expensive and can span multiple years, delaying the ability to adopt a new material and/or manufacturing methods or accept modifications to a previously established manufacturing process. While this is not necessarily unique to AM, the lack of comprehensive material property databases containing design allowable evaluations coupled with the perceived immaturity and lack of field experience has been cited as one of the main impediments to AM adoption [1, 2].

A material’s fatigue capability is generally characterized via mechanical testing. As with any material testing program, statistically significant sample sizes are desired to ensure results and decisions are not artificially influenced by small sample sizes. In other words, enough specimens and/or volume must be tested to provide a reasonable representation of the process and material capability, potentially capturing variation that may result from unintentional anomalies and statistically rare defects. While there is some industry guidance with regards to the amount of testing required to establish statistically significant sample sizes, particularly for static properties, there are no official documents that dictate the sample sizes of dynamic testing properties (e.g., fatigue) required to submit for qualification efforts. Such requirements are often driven by original equipment manufacturers (OEMs) internal documents and best practices and are related to the criticality of the components being considered. Thus, this article will focus on the use of a hybrid approach to augment and/or to compare to a baseline data set, previously referred to as a design allowable, rather than creation of the baseline itself.

While there are many ways to conduct fatigue testing, two common methods utilized in a safe-life regime, particularly in regulated fields, are: (1) strain-controlled fatigue per ASTM E606 [29] and (2) load-controlled fatigue per ASTM E466 [30]. Both assume axial-loading characteristics, typically on cylindrical cross-section samples. In general, tests are conducted via these methods to create a stress (S–N curve) or strain (ε−N curve) versus cycles to failure curve, representing high cycle fatigue or low cycle fatigue, respectively. In either scenario, this entails defining a specific set of test parameters, including temperature, test frequency, test stress/strain ratio, and stress/strain-life region of interest, as these variables can impact the scope and results of the test program. Ultimately, these S–N or ε−N curves may act as the nominal data to derive design allowables for designers to assess fatigue capability of components. It is important to perform enough tests to understand any nuances regarding the shape of these curves. If only a few stress/strain conditions are tested, there is a potential to miss transition points in material behavior (e.g., surface versus internal failures) [31–33]. The number of data points to generate a baseline is not standardized, although ASTM standards recommend no fewer than 10 tests be used per curve [29, 30]. Programs must weigh such considerations when generating fatigue design allowable curves—running tests at a few predefined stress/strain conditions focusing on variation or running tests at various stress/strain conditions focusing on establishing curve shape. Each approach has its own benefits and challenges, and the choice is typically based on internal practices and preferences.

**Fig. 1:**
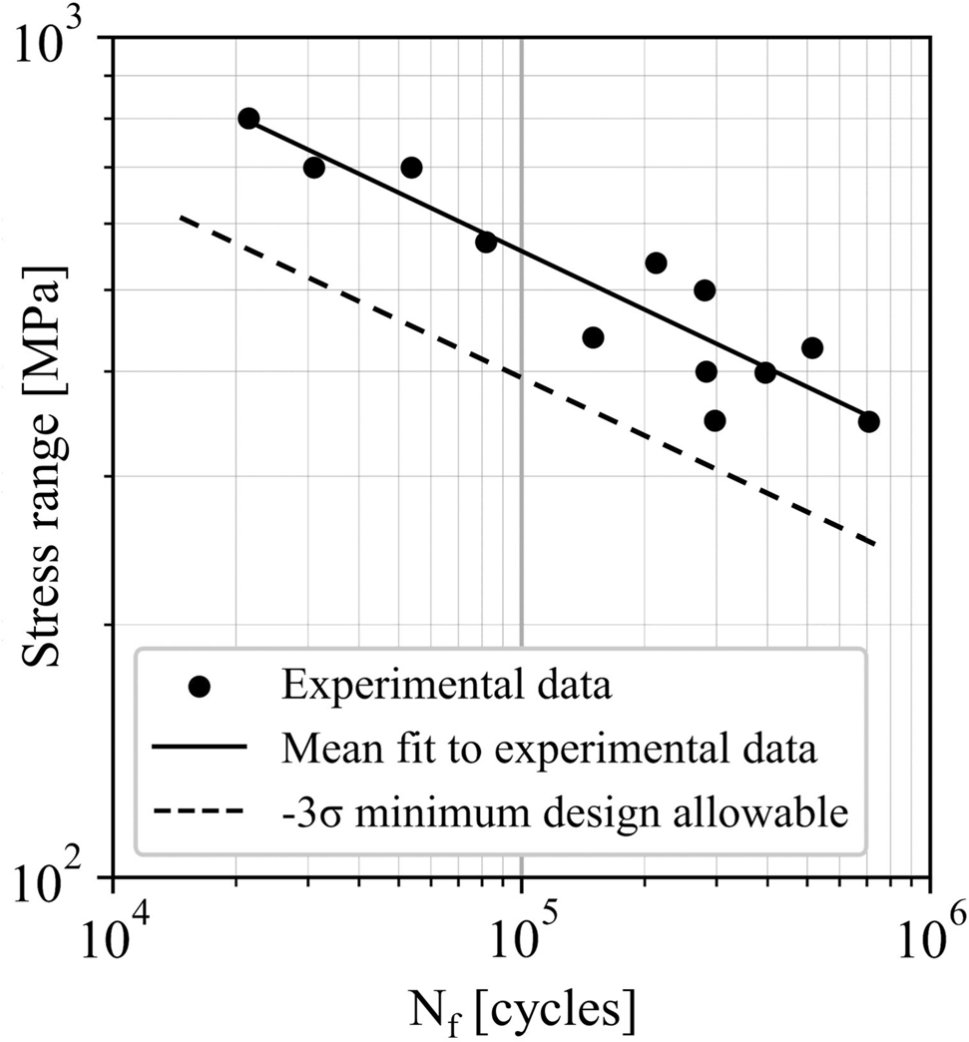
Additively manufactured alloy 718 data from Solberg et al. [36] is used to determine a nominal fatigue design allowable curve using the − 3σ minimum fit [34]

From these experimental tests, an average response and statistical minimum can be established. A regression based on a model, such as the Basquin or Coffin–Manson models, can be fit to the experimental data to provide an average response. The difference between the experimental data points and the predicted response from the regression establishes the residuals that can be used to calculate a statistical minimum. While there are many statistical minimum calculations that can be conducted, the most common ones referenced are based on standard deviations (*σ*) or confidence intervals around the average regression response. A − 3*σ* minimum represents a line offset from the average regression by three standard deviations, with the number of data points only influencing the standard deviation calculation [34]. The − 3*σ* minimum is computed by shifting the mean S–N curve in logarithmic coordinates to the left as follows:

1 $$ Y_{\min } \left( {X_{i} } \right) = \hat{Y}\left( {X_{i} } \right) - K \times s $$

where *X*_*i*_ represents the fatigue data in logarithmic coordinates, $\widehat{Y}$ is the regression curve of *X*_*i*_, *K* is a multiplier equal to 3, and *s* is the sample standard error of *Y* on *X*_*i*_. In practice, a distinction is made between the utilization of σ and *s* in Eq. (1) as follows. While σ characterizes the variation within a set of measurements (for example, cycles to failure at a given load), s measures the variation in means across multiple sets of measurements, encompassing the entire fatigue life curve [35]. On the other hand, a 95/99 minimum is based on confidence intervals, suggesting that there is 95% confidence that tests will fall above the defined curve 99% of the time [34]. Therefore, this calculation is heavily influenced by the number of tests included in the regression analysis. Figure 1 illustrates a S–N regression to experimental data to establish the mean fit as well as the -3*σ* minimum fit that represents the design allowable curve utilized to assess the fatigue life of a material [34]. Generally, fatigue design allowable curves are established to be the lower of either of these statistical calculations or to be an even more conservative curve based on engineering judgement. This is an important consideration when it comes to use of the hybrid approach to be discussed later in this document.

Traditional design allowables consider the baseline condition of the materials, which is difficult in the case of AM since many factors influence the material response. It is important to consider other variables that may drive variation of unintentional anomalies like porosity beyond the parameter set, such as powder lot, build orientation, build location (e.g., platform location/incidence angles, Z heights), and multi-laser assignment (e.g., platform location/incidence angles, laser alignment). While including all these variables in a test campaign creates a complex matrix to consider, it can increase the probability of characterizing material representative of the overall process to establish a robust set of design allowables while optimizing the number of tests required. In other words, testing fatigue bars created from a nominal set of process parameters may minimize porosity and could result in non-conservative design allowables for fatigue-limited applications.

To establish a robust set of design allowables, the question arises as to how many tests of potential off-nominal conditions may be required to statistically understand the impact of a factor such as porosity. In the case of AM, main variables may include things like orientation due to potential for anisotropic behavior and the machine and energy source used to print. Regarding this methodology article, an off-nominal condition used to generate a specific level of porosity/defects will be considered as a main variable. Thus, the number of off-nominal conditions to trial will dictate the amount of testing required to establish model inputs for the hybrid qualification approach. The next section will discuss porosity in AM in more detail, and section “AM Material Development and Characterization” discusses how such off-nominal conditions can be determined to seed desired pore defect characteristics, particularly if legacy DOE conditions are not known from parameter optimization trials.

## Typical Porosity in Additive Manufacturing

The existence of porosity in AM materials created via parameter settings is generally well-documented [13, 14, 37, 38]. For the sake of the approach considered, three types of porosity conditions will be discussed: (1) keyhole, (2) lack of fusion (LOF), and (3) linearly aligned/planar porosity, a subset of LOF and hereafter referred to as linear stitch.

### Pore Defect Nomenclature

**Fig. 2:**
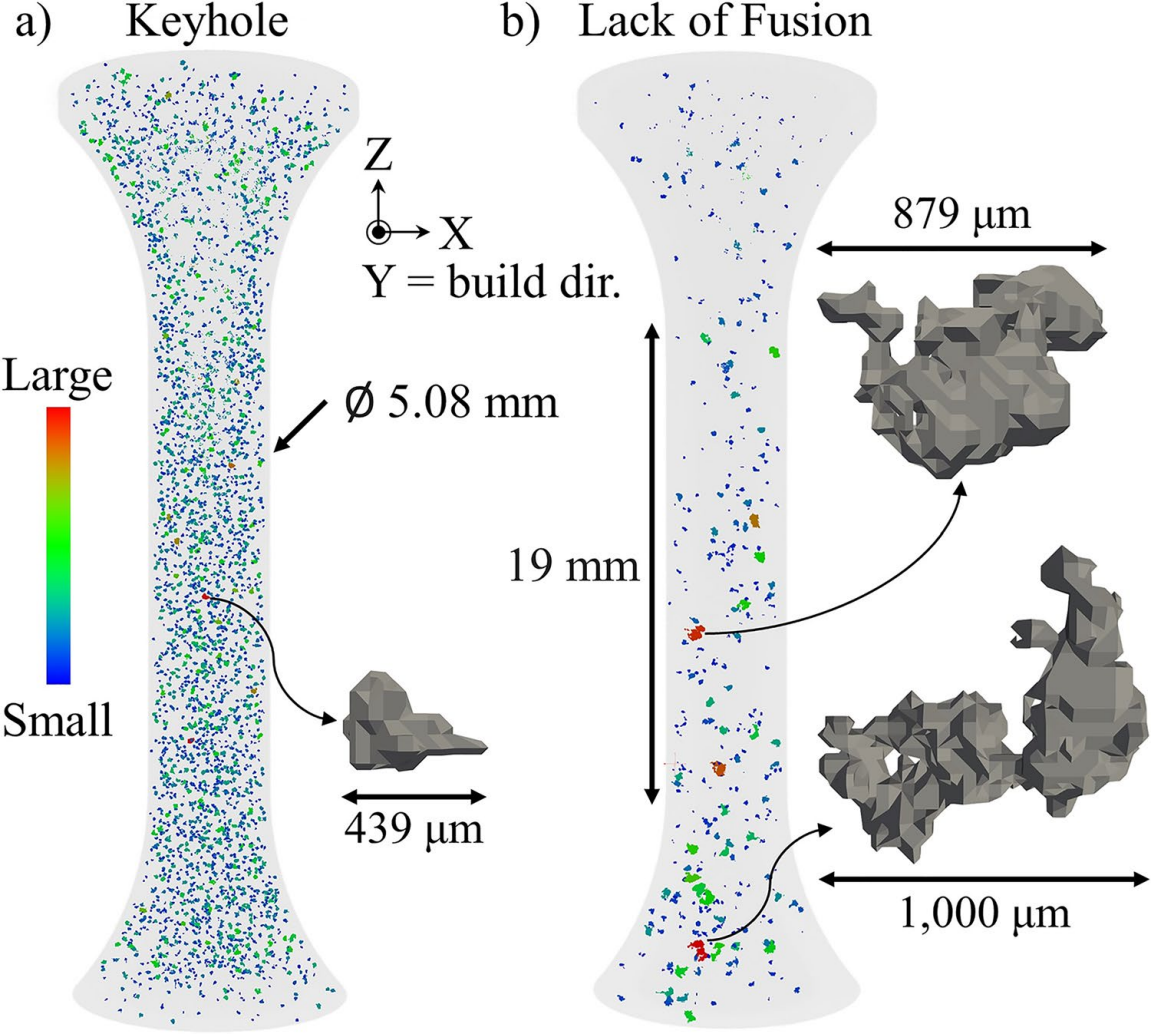
Examples of **a** keyhole and **b** lack of fusion pore defects detected using computed tomography in additively manufactured specimens of alloy 718. A keyhole pore and two of the largest lack of fusion pores are enlarged to emphasize the irregular morphology of the latter

**Fig. 3:**
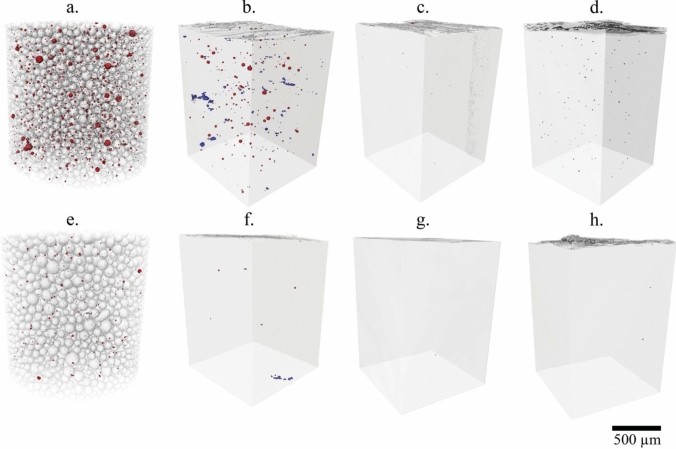
Micro-computed tomography (µCT) reconstructions depicting porosity in powders and coupons originating from AP&C (**a**–**d**) and TIMET (**e**–**h**). The scans are completed on: powder (**a** and **e**), as-built material (**b** and **f**), after hot isostatic pressing (HIP) (**c** and **g**), and HIP followed by heat treatment (**d** and **h**). Terms of Use: This figure comes from an open access article distributed under the terms of the Creative Commons CC BY license. It is attributed to Cunningham et al. [40], and the original version can be found here: https://doi.org/10.1080/21663831.2017.1340911

Keyhole porosity is created by the entrapment of process gas during melt pool formation, leading to spherical voids [13, 14]. This typically occurs when either the power is increased at a given speed setting or the speed is reduced at a given power setting, leading to increased energy input in a localized region. Furthermore, unless extreme, in theory, the impact on fatigue behavior would be less than the other types of porosity discussed below due to its spherical nature leading to less severe stress concentrations (see Fig. 2a [37, 39]). Additionally, there are other sources of gas porosity, due to the residual state of the powder. Cunningham et al. [40] demonstrated that porosity present in the powder from atomization, prior to the build process, persists throughout the subsequent processing of the material and results in a higher presence of porosity in the as built configuration and after heat treatment, as shown in Fig. 3.

Conversely, LOF porosity is created by the lack of energy input in a localized region [13, 14]. This can occur under multiple scenarios, including: (1) insufficient overlap between adjacent passes of the laser, or hatch spacing, (2) increased beam scanning speed with respect to the nominal parameter, and (3) reduced laser power with respect to the nominal parameter (e.g., potentially due to normal layer thickness variation during the build process). Generally, this type of porosity is the most prevalent “what if” scenario encountered during production where an optimized and stable parameter is being utilized. It can also significantly impact fatigue behavior due to the irregular pore morphology that can arise (see Fig. 2b).

Linear stitch porosity is a subset of LOF porosity created due to insufficient overlap spacing between adjacent passes of the beam [37]. This type of porosity is especially detrimental when the applied loading is orthogonal to the plane of pore defects and has the potential to cause an “unzipping” phenomenon as the crack front finds a path of least resistance through the aligned porosity [37]. This type of porosity is being explicitly discussed due to the increased interest in multi-laser systems. While linear stitch porosity could present itself in a single laser system for unoptimized parameters, this scenario is rarely observed. In a multi-laser system, the overlap is driven by the parameter controlling the laser paths as well as laser alignment.

### Non-destructive Evaluation

Pores are omnipresent in AM builds. Typically, AM materials produced with optimized parameters exhibit minimal bulk porosity, most of which are small (i.e., < 10 µm) and spherical. These types of pores are generally stochastic in nature and, thus, should be present in nominally processed test coupons. In geometric features of builds, such as overhangs, thin walls, and locations with sudden changes in heat conductivity paths, there is a greater probability of pore defects existing using the baseline bulk process parameters. Production level builds will often have witness coupons that may be destructively evaluated for both pore size distribution along with other metallurgical factors like grain size, but this is merely a snapshot of the build. If the coupon fails to meet material quality expectations, it automatically raises the question of the material quality of the entire build. Unfortunately, even if it passes expectations, it provides only directional assurance of hardware capability since the coupon does not represent the entire volume of the build or the geometric feature of the builds. Similarly, destructive mechanical testing of witness coupons is also limited in its ability to provide full assurance of material quality. Witness coupons are often based on tensile behavior being used as a proxy for more advanced and costly testing such as fatigue.

Non-destructive evaluation (NDE) provides qualitative and quantitative testing to determine the quality of the part within an acceptable degree of uncertainty. NDE can be used within a production setting to provide confidence for qualification, as well as quality control of the material builds. A recent review article by Quintana et al. [41] provides comparisons of NDE techniques for AM in terms of capabilities and recent advancements. As described by Quintana et al. [41], NDE can be categorized as: (1) visual testing, (2) ultrasonic testing (including laser ultrasounds and laser induced phase arrays), (3) acoustic emission, (4) electromagnetic testing (including eddy current testing), (5) radiographic testing (including tomography and micro-computed tomography), and (6) thermal and infrared testing. Of these techniques, ultrasonic inspection and radiography exhibit the best capabilities to detect porosity. Furthermore, several of these techniques offer promise for in operando use for detection of porosity, including in situ camera-based techniques, in situ laser ultrasounds, in situ radiographic monitoring, and melt pool monitoring via thermal or infrared measurements. There is tremendous promise in using these in operando sensing methods within a framework for rapid qualification and represents future work that can be used within this proposed framework standard.

Within NDE, there is a tradeoff between resolution and the size of the part in terms of penetration of the signal or field of view. One of the most common NDE methods employed for AM hardware is computed tomography (CT). CT equipment resolution can be tuned to detect very small voids in a material; this is often referred to micro-CT (µCT). To enable such resolution, however, the volume of the material evaluated may be limited with respect to the overall hardware size. Furthermore, the time required to scan an entire part at higher resolutions may require days instead of hours or even minutes, which would have a direct impact on part throughput and ultimately revenue. Hence, it is impractical to perform µCT on every part produced via AM.

Given the variation of pore defects across a geometric part, resolution limits of the NDE technique used, as well as the complexity, size, and material of the part, there exists a probability of detection for pore defects at each region in a part. Production level scans, depending on material and geometry, may only be able to reliably and repeatably resolve defects larger than ~ 250 µm (based on part sizes on the order of 100 mm in size) [42].

A significant portion of the pores are smaller than the detectable limits of existing, industrial NDE techniques, which requires new methodology. As such, the question arises as to whether the fatigue design allowable covers all scenarios of unintentional variation (e.g., porosity) that may exist in the material and that cannot be detected via NDE techniques. In other words: *what if the fatigue-limited/critical hardware has small, well-distributed porosity or porosity that is localized in a distinct geometric feature (i.e., not observed in the witness coupon) that is below the NDE detectable limit?* This document proposes an approach to increase confidence in nominal design allowables or produce augmented design allowables by reducing the reliance on empirical tests while addressing the possibility of unintentional variation in the form of porosity impact on material fatigue performance.

## AM Material Development and Characterization

As described in the section “Fatigue-Limited Parts and Design Allowables”, material qualification campaigns require many samples to adequately capture scatter and variability in the material response, particularly in fatigue-limited applications. This brute force approach is expensive and not conducive to large-scale production. As an alternative, the proposed approach described in this work is to intentionally seed the different abovementioned pore defects into AM builds directly and subsequently perform characterization, fatigue testing, and modeling. This reduces the number of coupons/specimens that must be built to evaluate the possible debit to fatigue resistance in the presence of stochastic pore defects.

**Fig. 4:**
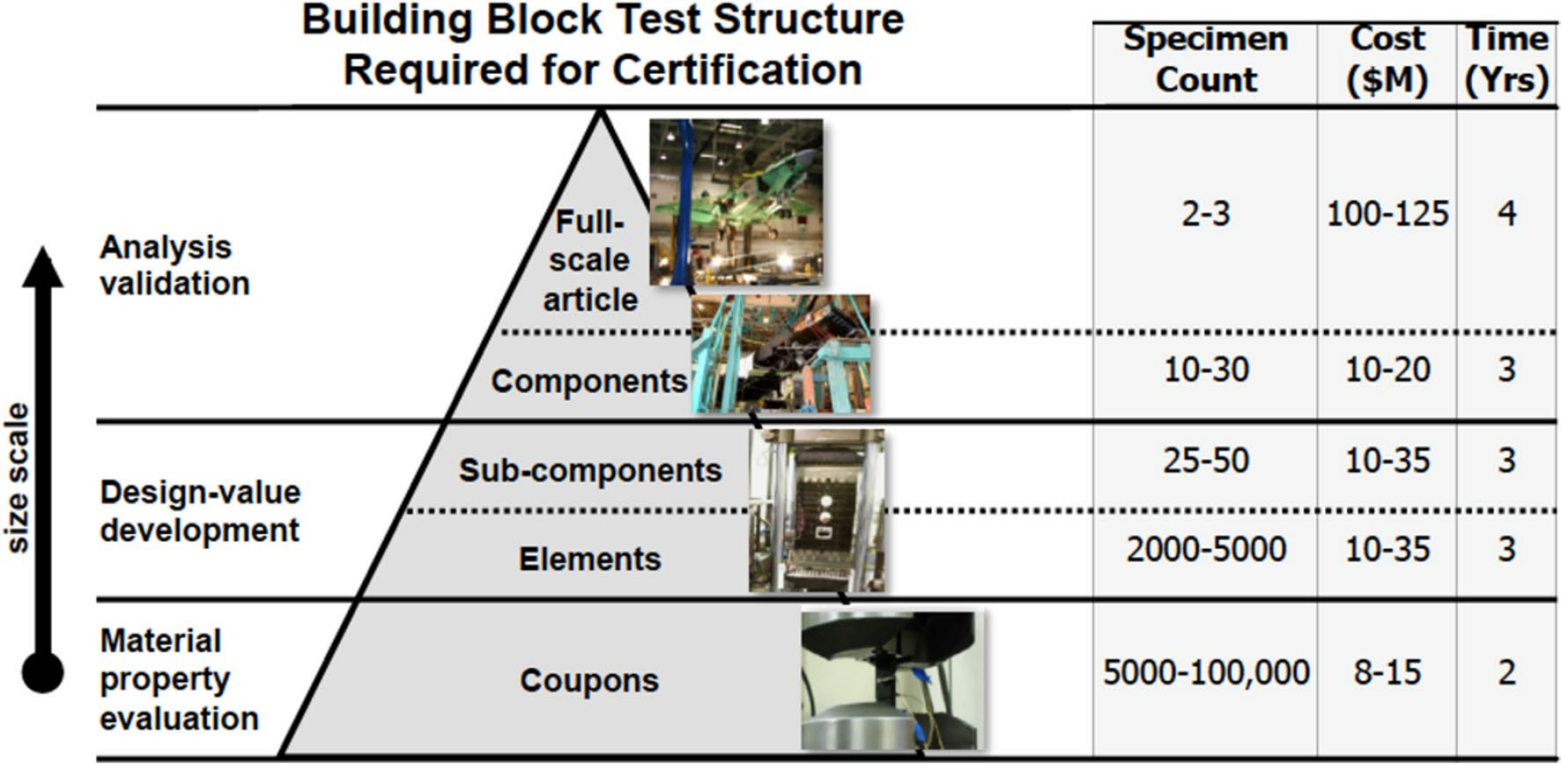
Example of a development pyramid. This image is courtesy of Mick Maher, used with permission, from Ref. [43]

In the context of qualification and certification, we examine the development pyramid illustrated in Fig. 4, which represents a typical certification test campaign and follows a bottom-up approach [43]. In this pyramid, initial testing begins with coupons to comprehend material properties, often involving tens of thousands of specimens. Testing progresses through elements, sub-components, components, and ultimately full-scale articles. As we ascend the pyramid, the specimen count decreases, but the cost, time, and test complexity increase. The framework proposed in this article aims to slenderize the development pyramid by reducing the number of specimens required during physical testing via the use of computational modeling, particularly near the bottom of the pyramid, thereby promoting a more rapid route toward qualification of AM.

**Fig. 5:**
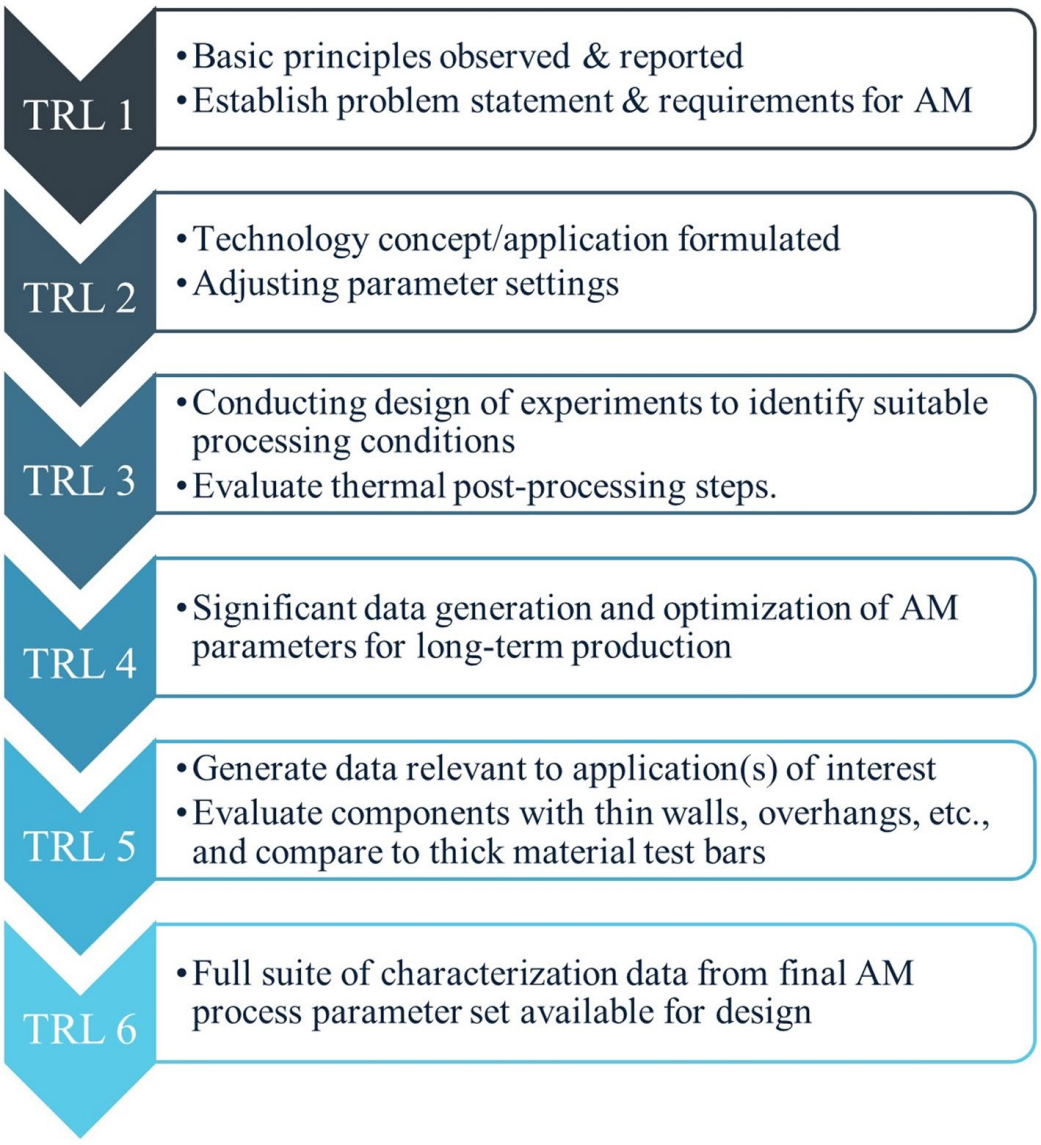
Technology readiness levels (TRLs) in the context of additive manufacturing

As with any new material and process development, following a structured approach ensures the appropriate evaluations occur as the process matures. One industry recognized approach developed by NASA is known as technology readiness levels (TRLs), which defines the overarching steps to consider while maturing a technology [44, 45]. While not specific to materials and process development, it provides a common language for collaborators to assess maturity. The relationship between the development of AM material and process to the TRL progression (i.e., TRL 1 through 6) is shown in Fig. 5.

### Parameter Optimization to Establish Baseline Condition

Prior to intentionally seeding defects in AM material, the baseline condition for processing should be established. In the field of AM, parameter optimization may be interpreted differently based on the part requirements. As in any material development process, there are often tradeoffs that need to be considered. For example, some parts may require high strength, but this may be at the expense of ductility. When it comes to fatigue-limited parts, AM processes are developed to minimize the amount and types of porosity as described above. Depending on part requirements, there may be tradeoffs with regards to the amount of productivity that can be achieved while maintaining acceptable porosity and as-built microstructure to meet material property requirements. Thus, the first step (i.e., TRL 2) in parameter optimization is to collect and prioritize requirements to create the design of experiment (DOE) conditions for consideration (e.g., viable layer thickness, laser power based on machine architecture, etc.). Additionally, theoretical calculations of AM build speeds may narrow the range of the DOE conditions based on a potential business case required to justify pursuing AM for material qualification and part certification efforts. These productivity needs should be included as part of the DOE conditions. For example, theoretical calculations may indicate that the layer thickness may be at most 100 µm if the machine architecture can only achieve a maximum power of 400 W. Since this guideline addresses bulk material capability, the following discussion will focus on only a piece of the parameter development that needs to occur as part of an overarching parameter development effort; contour/surface finish development will not be addressed as part of this effort but will be mentioned in the “Application of the Hybrid Methodology for Zone-Based Life Analysis” section.

Optimal parameters are often established as part of TRL 3 and 4 efforts via a series of DOEs, which typically consider porosity, microstructure, tensile strength, and ductility. In general, there are five main factors that may be varied to achieve optimal porosity and as-built microstructure: (1) laser power, (2) laser spot size, (3) laser speed, (4) hatch spacing, and (5) powder layer thickness. Based on these conditions, a multi-factorial DOE may be constructed using some or all of the five variables to establish viable process windows based on a given criteria. Porosity is often used as a first level assessment criteria on the DOE parameters. Multiple DOEs may be necessary to narrow a process window, particularly when factoring in post-processing steps necessary to optimize the microstructure and mechanical properties. For example, a parameter with acceptable porosity may have an as-built microstructure that is less likely to convert to an equiaxed grain structure after thermal processing. This is often why a phased approach may be necessary to develop a fully optimized process; it is not only a function of machine parameters.

Once a parameter (or series of parameters) and post-processing steps are down selected based on porosity and microstructure, it is appropriate to conduct more time and cost intensive testing, including fatigue. Typically, due to cost, only viable baseline parameters are typically tested as part of the TRL 3 and beyond. However, in the context of this guideline, understanding the non-optimal or off-nominal parameters (i.e., increased porosity levels) and their impact on fatigue behavior can benefit the hybrid approach presented to establish more robust design allowables.

### Seeding Different Types of Pore Defects

The pores described in the section “Typical Porosity in Additive Manufacturing”, even with size below typical production NDE limits (~ 250 µm for production components on the order of 100 mm in size [42]), may notably debit the fatigue resistance of AM components. A methodology to seed these pores using a DOE is discussed in Ref. [37]. This methodology relies on an established baseline parameter set that produces near optimal builds with minimal porosity. AM process parameters are then systematically perturbed to achieve the desired pore characteristics such as average and maximum pore size and porosity volume fraction [37].

The primary process parameters are often collapsed into a single parameter called the laser energy density (LED), computed as

2 $$ {\text{LED}} = \frac{P}{vht} $$

where $P$ is laser power, $v$ is scan speed, *h* is hatch spacing, and *t* is layer thickness. Several other parameters influence the presence of pore defects and include number of samples, location, orientation on build platform, laser spot size, layer-to-layer rotation, process gas flow, machine used, etc. However, the focus here is on the primary processing parameters in Eq. (2). As described in the section “Typical Porosity in Additive Manufacturing”, keyhole porosity typically occurs through an increase in LED. The normalized enthalpy, defined as the ratio of laser input enthalpy, $\Delta H$, to the enthalpy of the material at melting, ${h}_{s}$, can also be used to predict melt pool morphology and monitor the transition of a stable melt pool to the formation of keyhole porosity. The normalized enthalpy is computed as

3 $$ \frac{\Delta H}{{h_{s} }} = \frac{AP}{{\pi \rho C_{p} T_{m} \sqrt {Dva^{3} } }} $$

where $A$ is the absorptivity, $\rho$ is density, ${C}_{p}$ is specific heat capacity, ${T}_{m}$ is the melting temperature, $D$ is thermal diffusivity, and $a$ is the laser spot size. The normalized enthalpy provides further control over the formation and embedding of keyhole pores since it considers the absorptivity, which has been shown to vary with melt pool properties such as temperature and the spot size [46–48]. In contrast to keyhole pores, the formation of LOF pore defects is due to inadequate local energy input. The predicted width of the melt pool can be computed as

4 $$ w = \sqrt {\frac{8P}{{e\pi \rho C_{p} \left( {T_{m} - T_{0} } \right)v}}} $$

where ${T}_{0}$ is the spatially uniform initial temperature of the substrate and *e* is the exponential constant. Along with the hatch spacing, the beam power and scan speed can be altered to decrease the melt pool overlap between adjacent passes of the beam and to systematically control the formation of LOF pores.

**Fig. 6:**
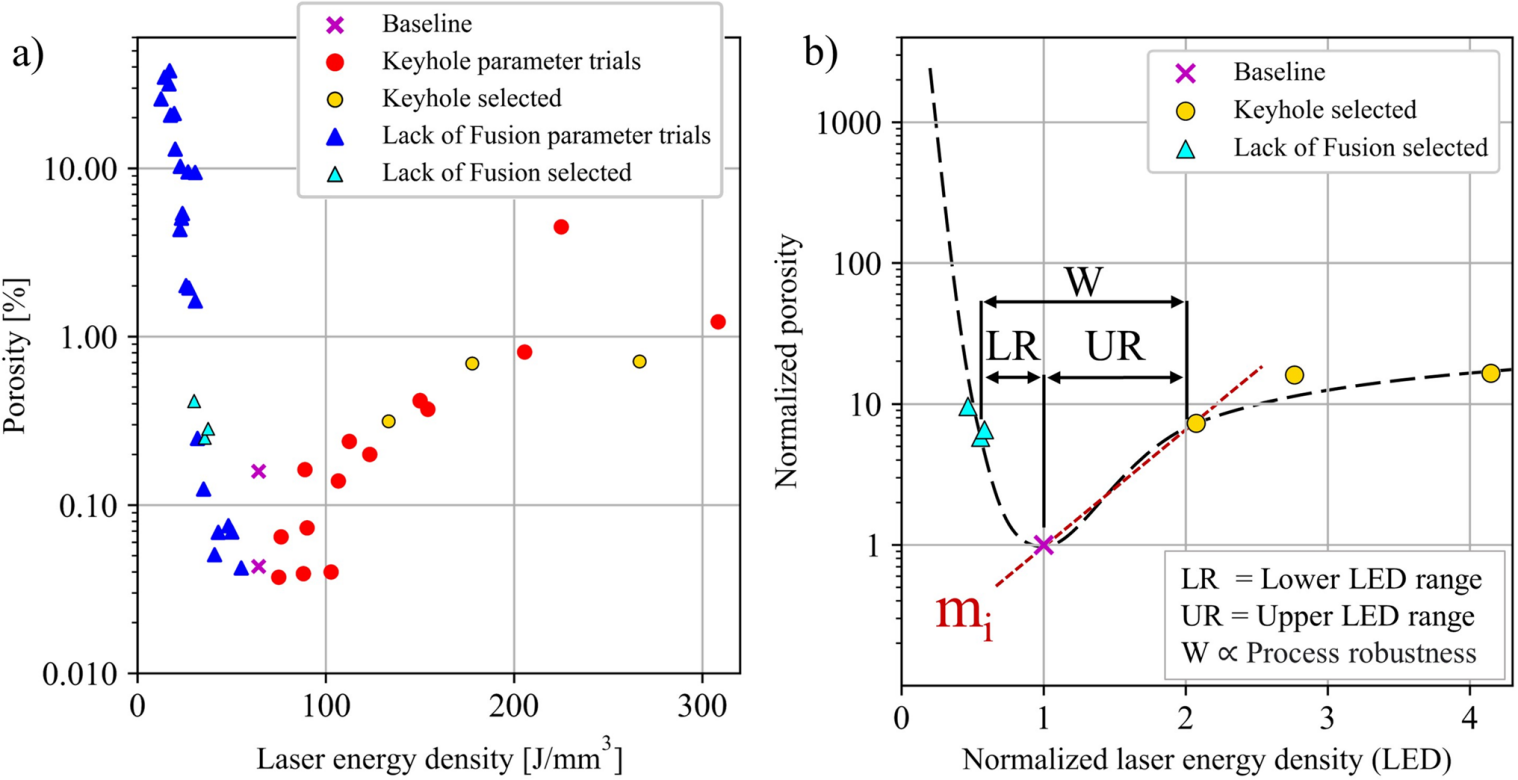
Design of experiments (DOE) to determine process parameters to intentionally seed keyhole and lack of fusion pores in additively manufactured builds of alloy 718. **a** Porosity volume fraction as a function of laser energy density. **b** Normalized porosity as a function of the normalized laser energy density. The selected parameter trials provide insight into the robustness of the process parameters. The normalized process slope m_i_ referenced in Eq. (5) is estimated in (**b**)

The methodology described here was recently demonstrated using AM builds of alloy 718 [37]. Keyhole and LOF pores were seeded with a target porosity volume fraction between 0.4 and 1%, determined such that the porosity can notably perturb the fatigue response but does not impact specimen ductility or bias the fatigue crack formation site due to a smaller effective cross-sectional area. Additionally, the target maximum pore size was 400 µm to evade detectability using production NDE techniques [42]. AM builds were sectioned, and pore defects were characterized using optical microscopy. Figure 6a depicts porosity volume fraction in the AM builds as a function of LED. The selected process parameters are highlighted in Fig. 6b in which the LEDs and porosity volume fractions are normalized by the baseline parameter set, i.e., the parameter set that minimizes porosity. A decrease in LED results in a steep rise in porosity and indicates a sensitive response to process parameters. On the other hand, an increase in LED results in a gradual increase in porosity. To summarize, for a near-optimum starting process parameter set for alloy 718, the following conditions were achieved as shown in Fig. 6:

- 10 × increase in LOF porosity (lower LED boundary, LR) from the porosity values in the baseline condition at a parameter set of 0.5 × Baseline LED value
- 10 × increase in keyhole porosity (upper LED boundary, UR) from the porosity values in the baseline condition at a parameter set of 2.0 × Baseline LED value

For a general case with limited data, a 10 × increase in porosity can be achieved by changing the LED as

5 $$ \Delta {\text{LED}}_{{{\text{Normalized}}}} \cong \pm \frac{10}{{m_{i} }};\,\,m_{i} = {\text{ normalized}}\,{\text{ process' }}{\text{slope}}$$

where the normalized process slope ${m}_{i}$ is estimated from Fig. 6b.

**Fig. 7:**
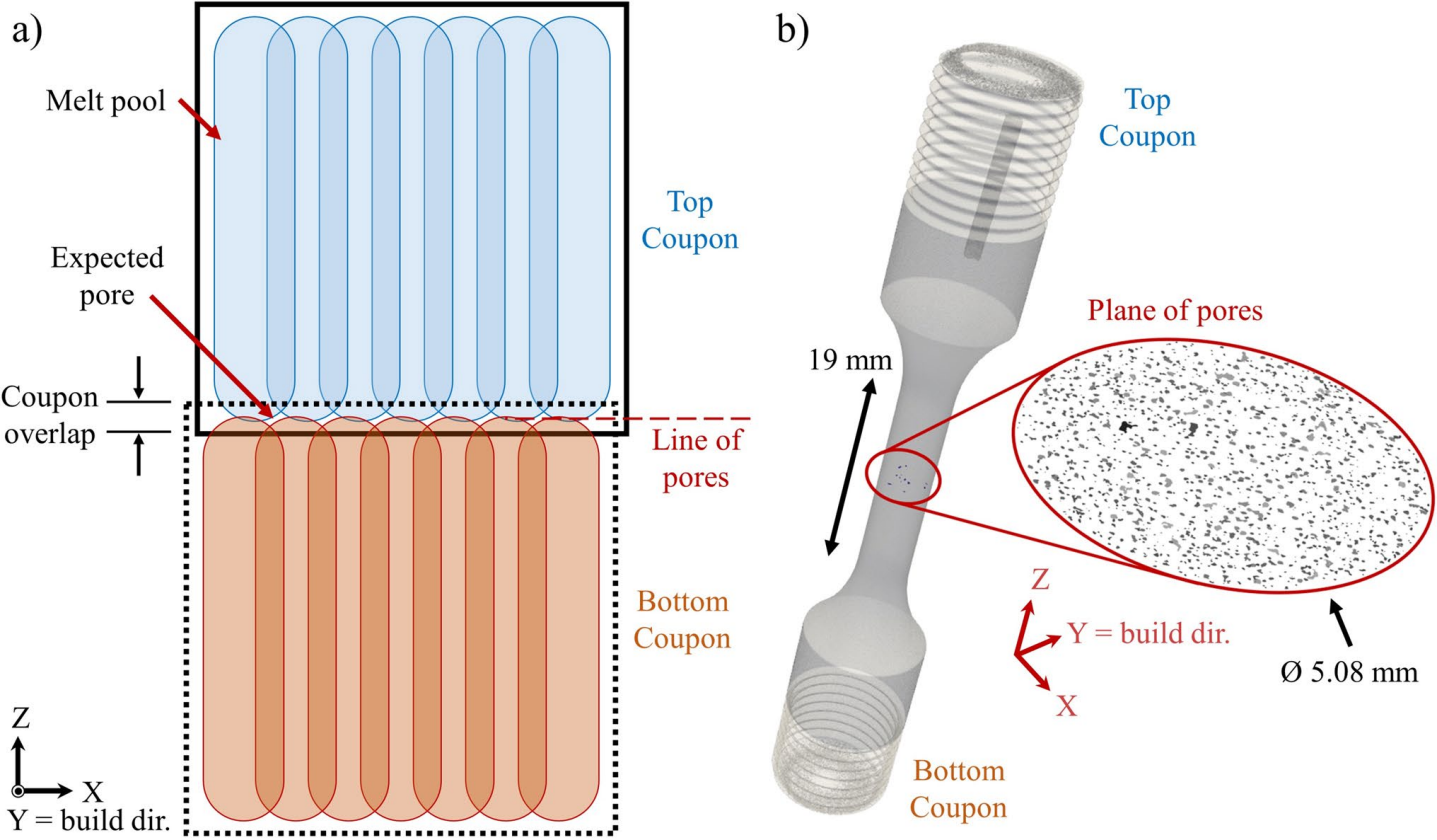
**a** Schematic of a single layer of the controlled geometric approach to seed the linear stitch porosity condition in additively manufactured builds. Note that only a single laser scan orientation is depicted here. As the part is built and the laser scan path is rotated, the lines of pores coalesce into a plane. **b** Computed tomography scan of a machined fatigue specimen with a plane of pores seeded perpendicular to the loading direction

A schematic of the linear stitch porosity condition is shown in Fig. 7a, in which a controlled geometric approach is employed with two computer aided design (CAD) models to induce linear aligned/planar pore defects [37]. The coupon overlap between the two CAD models controls the degree of porosity and no outer contour is applied in this build configuration which would prevent this porosity condition. Figure 7a depicts a single scan layer in which the tips of the melt pools do not completely overlap and produce a line of pores. As subsequent layers are built and the laser scan path is rotated, the lines of pores coalesce into a plane. Figure 7b depicts a CT scan of a machined fatigue specimen with a plane of pores seeded perpendicular to the loading direction using this controlled geometric approach [37].

### Seeding Pores Without Changing the Surrounding Microstructure or Mechanical Strength Behavior

The methodology to seed representative pores relies on the ability to not significantly alter the microstructure or mechanical strength behavior of specimens, in terms of yield point and work hardening response, as this may skew the analysis of fatigue experiments. AM specimens built using different process parameters should thus be characterized after the application of any post-processing heat treatments, which include stress relief, homogenization, solution treatment, and aging for alloy 718 [49].

Electron backscatter diffraction (EBSD) can be employed to examine the microstructure of AM builds. Metrics such as grain size and the presence of crystallographic texture can then be compared between different specimens to determine whether statistically significant differences exist [37]. Additionally, different material systems may provide additional metrics that can be compared between specimens built using different process parameters, e.g., length fraction of annealing twins, volume fraction of different phases in multiphase alloys, etc.

**Fig. 8:**
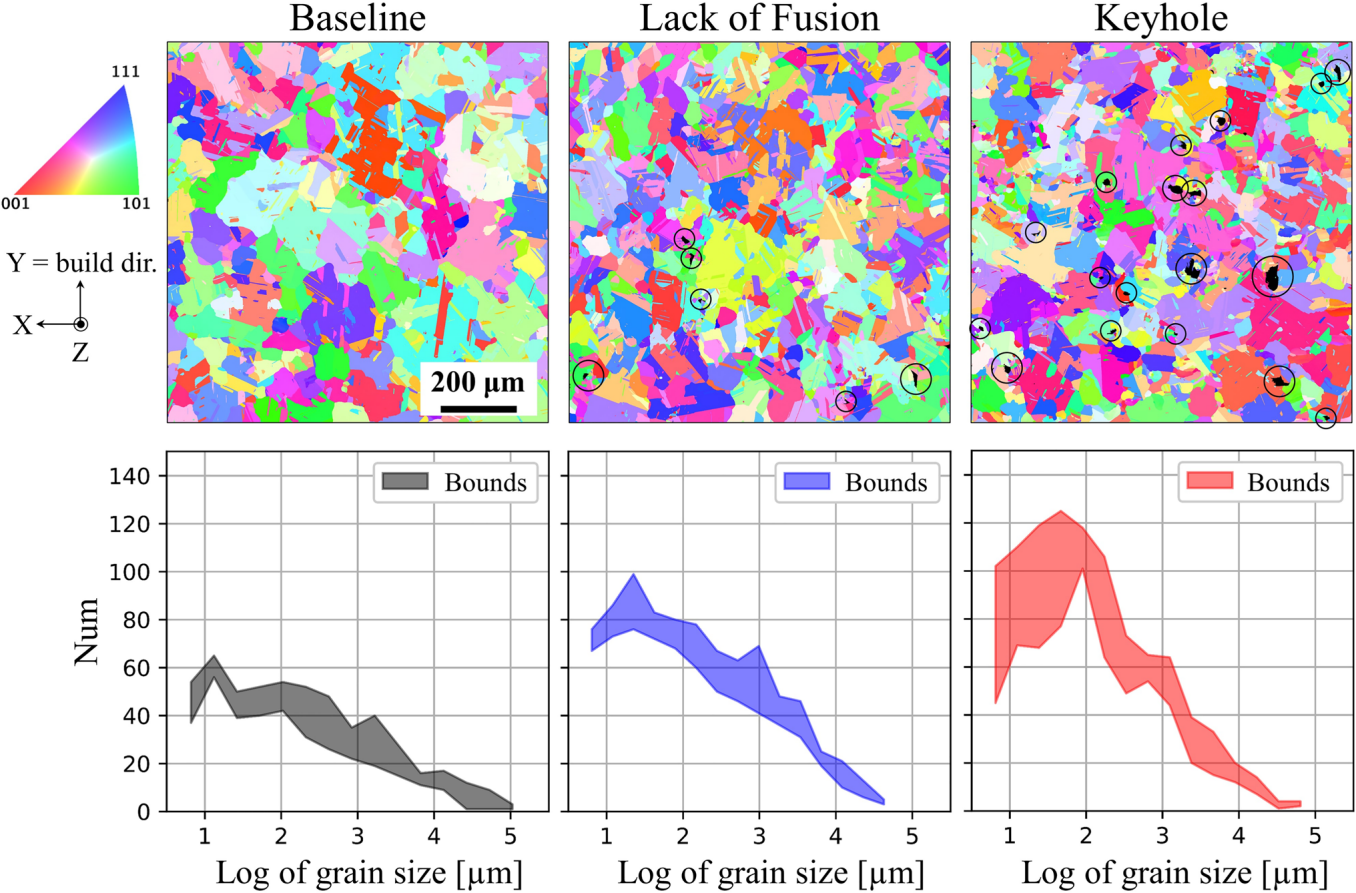
Comparison of electron backscatter diffraction (EBSD) scans of additively manufactured alloy 718 specimens built with a baseline process parameter set (i.e., parameters to minimize porosity) and process parameters identified to seed lack of fusion and keyhole pores, which are circled in the two latter conditions. The EBSD scans are depicted as inverse pole figure with the reference direction in the Z direction. The EBSD scans are split into quadrants from which grain size distributions are computed, the bounds of which are shown in the bottom row

Figure 8 depicts EBSD scans of AM alloy 718 specimens built using a baseline (i.e., control) process parameter set alongside specimens built using process parameters identified to seed lack of fusion and keyhole pores [37]. The specimens underwent a heat treatment that included stress relief (1.5 h at 1065 °C), homogenization treatment (1 h at 1177 °C), solution treatment (1 h at 982 °C), and aging (8 h at 718 °C followed by 18 h at 621 °C) [37]. EBSD would ideally be conducted on multiple specimens from each condition to develop a statistical assessment. The EBSD scans had a resolution of 0.5 μm/pixel with a 2000 × 2000 pixel or 1 mm^2^ field of view and were subsequently split into quadrants and the grain size distribution in each quadrant is analyzed using the MTEX software [50] with a 5° grain misorientation tolerance and a minimum grain size of 12 pixels. Bounds of the grain size distributions are shown in the bottom row of Fig. 8 and demonstrate variability within individual EBSD scan, particularly for the keyhole condition. Nonetheless, the bounds of the grain size distributions overlap between the three specimens, indicating similarity in microstructure. Furthermore, crystallographic texture is random in each specimen, i.e., grains are not preferentially aligned.

**Fig. 9:**
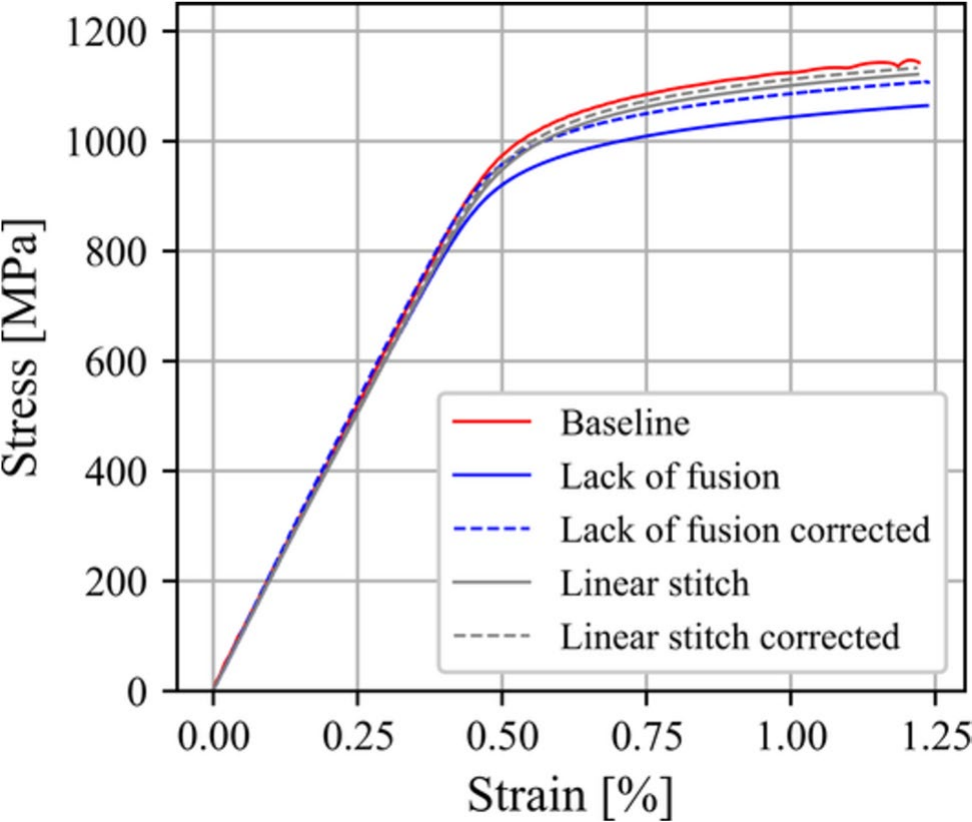
Macroscopic stress–strain curves for specimens seeded with the lack of fusion and linear stitch porosity conditions alongside a specimen built with nominal process parameters, i.e., minimal stochastic porosity. Pores in the lack of fusion and linear stitch specimens are visualized in Figs. 2b and 7b, respectively. The measured stress for these two specimens was corrected by considering the maximum projected area of pores normal to the loading direction

Potential differences in microstructure can additionally be assessed using the macroscopic stress–strain response. Figure 9 compares the monotonic response of a baseline specimen and specimens seeded with the lack of fusion and linear stitch porosity conditions, the latter two of which are visualized in Figs. 2b and 7b, respectively. The nominal conditions report stress as the applied load divided by the original cross-sectional area of the specimen, as determined via caliper measurements after machining. Since these two latter specimens contain considerable porosity, their measured stress response was modified by considering a corrected cross-sectional area. For the corrected cross-sectional area, the maximum projected area of pores perpendicular to the loading direction was identified via X-ray tomography [37]. As shown in Fig. 9, by correcting the cross-sectional area of the LOF and linear stitch specimens, the stress–strain curves are consistent with the baseline specimen, which demonstrates the mechanical strength (yield and hardening behavior) of the material was not affected by intentionally inserting pore defects. Moreover, the corrected stress–strain curves in Fig. 9 conform to the scatter observed in other baseline specimen stress–strain curves.

## Fatigue Modeling

Many models have been proposed to account for the role of porosity in additively manufactured materials, including phase field modeling [51], linear elastic fracture mechanics [12], cohesive zone modeling [52], peridynamics [53], and probabilistic approaches [54–56]. As an illustrative example, crystal plasticity is discussed for use within a finite element solver. Crystal plasticity finite element (CPFE) modeling accounts for the elastic and plastic anisotropies at the grain level, as well as the ability to mesh around porosity and microstructural features. This approach uses a multiplicative decomposition of the deformation gradient into an elastic and plastic component, in which the velocity gradient is determined by the shear strain rate induced by crystallographic slip, accounting for the kinematics of the slip systems, in each element. The result of this framework enables the calculation of the stress concentration and plastic strain accumulation near defect and microstructural features that can lead to damage and overall failure of the material. This microstructure-sensitive CPFE framework is demonstrated for the.

**Fig. 10:**
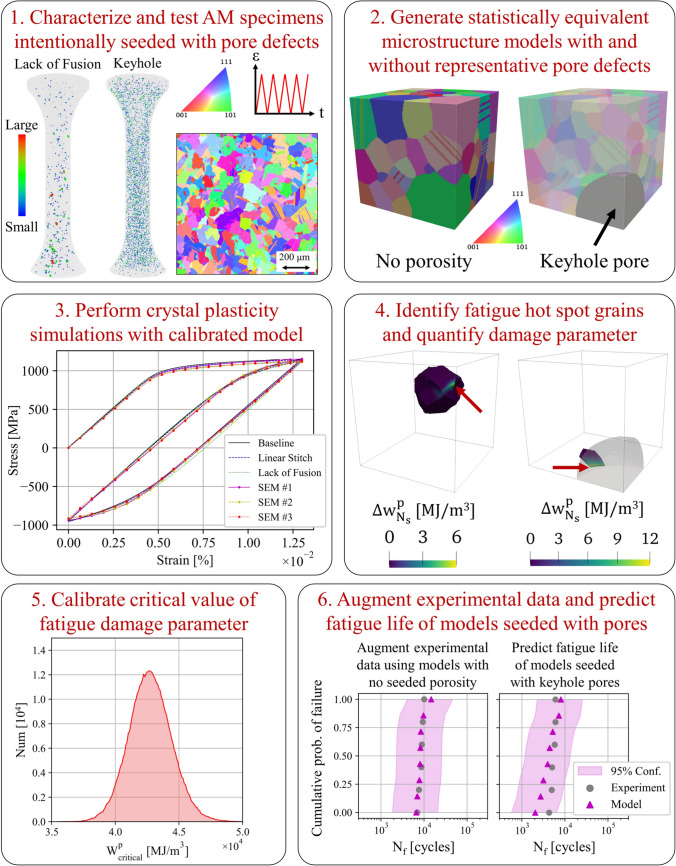
Overview of the microstructure-sensitive fatigue modeling framework [37, 39]. Additively manufactured specimens with intentionally seeded pore defects are characterized using computed tomography, electron backscatter diffraction, and fatigue testing. Statistically equivalent microstructure models undergo crystal plasticity simulations and fatigue hot spots are identified. A fatigue damage parameter is calibrated using experimental data and subsequently used to predict fatigue lives of models

The general approach described in this paper can be used with any modeling approach that accounts for local porosity and microstructural defects and is not specific to crystal plasticity modeling. Due to past experience, the authors have selected crystal plasticity as the modeling strategy used for the hybrid modeling and experimental approach toward rapid qualification and certification. The crystal plasticity theory and governing equations will not be discussed in this paper, and the reader is referred to [57] for details. An overview of the microstructure-sensitive modeling framework is shown in Fig. 10 and will be discussed in the subsequent sub-sections.

The requirements for microstructure-sensitive modeling are (1) a calibrated constitutive model (as discussed in the next subsection) and (2) digital microstructure models that capture the relevant microstructure features, e.g., grain size distribution, pore defects, annealing twins, multiple phases, etc. and that serve as inputs to the constitutive model. Open-source software packages such as NEPER [58, 59] and DREAM.3D [60] are vital in generating realistic microstructure models for simulation. These packages can generate statistically equivalent microstructure models using data from previous characterization, e.g., grain size, twin length/area fraction, crystallographic texture, etc., and reconstruct experimental data sets, e.g., 2D models from EBSD or 3D models from serial sectioning and EBSD or high-energy x-ray diffraction microscopy (HEDM) [59–61]). They also integrate well with open-source crystal plasticity software packages [62–66].

Several methods exist to embed pore defects into digital microstructure models. Spherical or ellipsoidal pores, with size and spatial distributions characterized experimentally or predicted using computational models, can be generated as separated phases in the digital models that are then removed from the mesh before simulation. The complex morphology of LOF pores can similarly be characterized experimentally or predicted computationally and subsequently overlaid onto fully dense models to create representative pore models [39].

An important consideration in the development of computational models is the associated maturity level, akin to the TRLs discussed previously. These model maturity levels (MMLs) were described in the context of ICME models in Refs. [67, 68]. For a comprehensive understanding of MMLs, including descriptions, categories, and criteria considerations, readers are directed to Table 2 and 3 in Ref. [67].

### Mechanical Test Data, Fatigue Data, and Model Calibration

In order to parameterize the microstructure-sensitive fatigue model, calibration is needed against experimental data. The necessary experimental data are summarized as follows: (1) single crystal elastic constants, (2) large deformation hysteresis loop evolution during cyclic loading, and (3) fatigue experiments, measuring cycles to crack initiation. Each of these experiments should be conducted at the temperature(s) of interest for the model, which requires repeated testing across a range of temperatures to create a model that is applicable at more than one temperature.

First, to account for grain-level anisotropy, single crystal elastic constants are required, which is challenging as it requires processing single crystals of complex engineering alloys or inferencing methods based on local texture and micromechanical measurements [69–71]. Since these are challenging measurements, single crystal values can be acquired from literature, although care should be taken to determine the precision and method to acquire such values.

To calibrate the role of plasticity, large deformation testing of the material is needed to establish initial yield and hardening behavior. This can be accomplished via full stress–strain curves under monotonic loading or cyclic loading ensuring the deformation is sufficiently large to create an open hysteresis loop behavior, e.g., macroscopic plasticity is observed in the initial loading cycles. Ideally, the hysteresis loop behavior should be repeated at several loading ratios (R-ratios) and if the material is tested in a strain-rate sensitive regime, multiple strain rates should be employed. During calibration, identifying the appropriate crystal plasticity parameters to capture the experimental data does not result in a unique solution. As mentioned above, additional experimental tests over a range of conditions (R-ratios, strain rates, temperatures, repeat tests, etc.) are preferred, but are accompanied by additional time and cost and present complexity for parameterizing the model. Over the years, the calibration of the crystal plasticity has been accomplished via optimization routines of macroscopic behavior [72, 73] or direct comparisons to micromechanical, crystal-level fields [74, 75]. Lastly, within cyclic loading, capturing the appropriate yielding upon reverse loading is critical to predict the fatigue behavior. As a consequence, accurate representation of the backstress formulation and evolution is important to fatigue modeling [76, 77].

A set of fatigue crack initiation experiments should be conducted, either tested in high cycle fatigue (load controlled) or low cycle fatigue (strain controlled), based on the applicable usage of the intended model. A sufficient number of specimens should be tested to quantify variability or scatter in the fatigue results, for instance at least 10 specimens per loading condition or S–N/ε−N plot. A regression fit can be applied to the S–N or ε−N data, for example using the Basquin or Coffin–Manson power-law, respectively, whereas the residual between the individual data points and the regression can also be used to assess the degree of scatter in the fatigue behavior. Afterward, the fatigue metric, discussed in more detail in the next subsection, can be calibrated against the experimental data. Lastly, it is beneficial to perform fractography on the failed specimens, to identify the cause of crack initiation, porosity (including measuring the dominant pore size), free surface, or crystallographic facet. The cause of crack initiation is useful to compare to model predictions, to ensure the correct mechanism of crack initiation is captured by the model [78].

### Fatigue Model Predictions

Using a microstructure-sensitive model, the fatigue life distribution can be predicted for each set of seeded pores. Each virtual microstructure is generated from the statistical distributions of pores and grain level features, such that each virtual microstructure is distinct, but the distribution of features is statistically similar to prior simulations. From the simulations, the local stress concentrations and accumulated plastic strain during cyclic loading are calculated as a function of loading cycles. In the literature, there has been many metrics proposed over the years to capture crack initiation or advancement of small fatigue cracks, including the Fatemi–Socie parameter [79, 80], stored energy density [81], and wedge-crack model via dislocation pile-up [82]. Each of these models have shown promise in their prediction of crack initiation and are complementary in their construction. In this work, the critical accumulated plastic stored energy density (APSED) is used. Based on the authors’ experience and past validation efforts, the APSED metric has been identified as a single metric to predict crack initiation, which can be used as a material parameter across applied loading states or in the presence of porosity [23]. It is an accepted practice to measure these fields during cyclic loading, until the change in the metric’s value per cycle has saturated, at which point shakedown has occurred in the fatigue simulation, and the results can be forward extrapolated based on a linear fit. The series of linear projections for each virtual microstructure can be compared to the series of experimental tests, in which Bayesian inference methods can be used to calibrate the appropriate critical value of the APSED [23]. The advantage of the Bayesian approach is that it can be updated based on future experiments and is amenable for direct inferencing of the uncertainty in the fatigue metric values. The APSED is determined for the baseline process parameters, which is consistent across all simulations regardless of loading or the presence of porosity. When seeded porosity is included in the model, the fatigue predictions exhibit a debit in their expected life. Hence, the output of such a model is the expected fatigue life debit for each loading condition, for each group of seeded pore defect distributions, as discussed in the section “Typical Porosity in Additive Manufacturing”.

In order to build trust within the fatigue model predictions, appropriate levels of verification, validation, and uncertainty quantification must be completed. Verification refers to the computational model’s implementation accurately capturing the mathematical framework and solution. The reader is referred to [67] for more information. Validation refers to the model capturing the appropriate physics, both in terms of the formation of the model and the parameterization of any model values. For the present fatigue model, this can be accomplished in several steps. First, the coupon level probability of failure can be compared to the macroscale probability of failure for different loading conditions to ensure the critical APSED captures the fatigue behavior [23, 83]. The appropriate range of fatigue data should be log-normal in nature, exhibit increased scatter at lower applied loads, capture mean behavior as well as the tails of the distribution. Moreover, to ensure the model captures the appropriate physics, a direct comparison to local behavior is preferred. A direct comparison is such that the model provides a one-to-one recreation of the physical microstructure and defects of the material, such that the micromechanical fields and cracking phenomenon can be unambiguously compared throughout their loading evolution. The advantage of such an approach is the model can be assessed at the appropriate length-scale, since the model and experiment exhibit similar length scales of their measurement, compared to coupon level tests, in which the model behavior is averaged across several length scales. An example of such a direct means of comparison is shown in Ref. [84], where a microstructure-sensitive fatigue model predicts the location of fatigue crack initiation consistent with the companion experiment. These examples demonstrate validated implementations of CPFE models with reduced calibration data bias [67]. Additionally, the digital microstructure models used to populate the fatigue response are independent of the experimental specimens used for calibration data collection, although their distributions of microstructure features (e.g., grain size distribution, area fraction of twins, etc.) are statistically similar.

Further uncertainty in the parameterization of the underlying crystal plasticity model should be assessed, to determine how the reliability in determining individual crystal plasticity parameters result in discrepancies in the calculated quantities. Due to the high computational cost in running crystal plasticity models, it is time consuming to conduct such rigorous uncertainty quantifications, yet still necessary. Several recent studies have examined these types of uncertainties in the crystal plasticity model parameterization [73, 85, 86]. With the model form of the constitutive equations used in the flow rules and hardening equations for crystal plasticity, opportunities exist to include more physics-based relationships, which often come with additional parameters. The inclusion of these additional parameters provides the opportunity to produce a better fit with experimental data, e.g., additional degrees of freedom for calibration, yet with the additional parameters also comes additional sources of uncertainty. Calibrating the critical value of the fatigue metric can also produce uncertainty, which can be quantified using Bayesian analysis. As shown in Ref. [87], considering each of these forms of uncertainty, one can propagate the uncertainties to provide a confidence bounds for the fatigue life predictions.

**Fig. 11:**
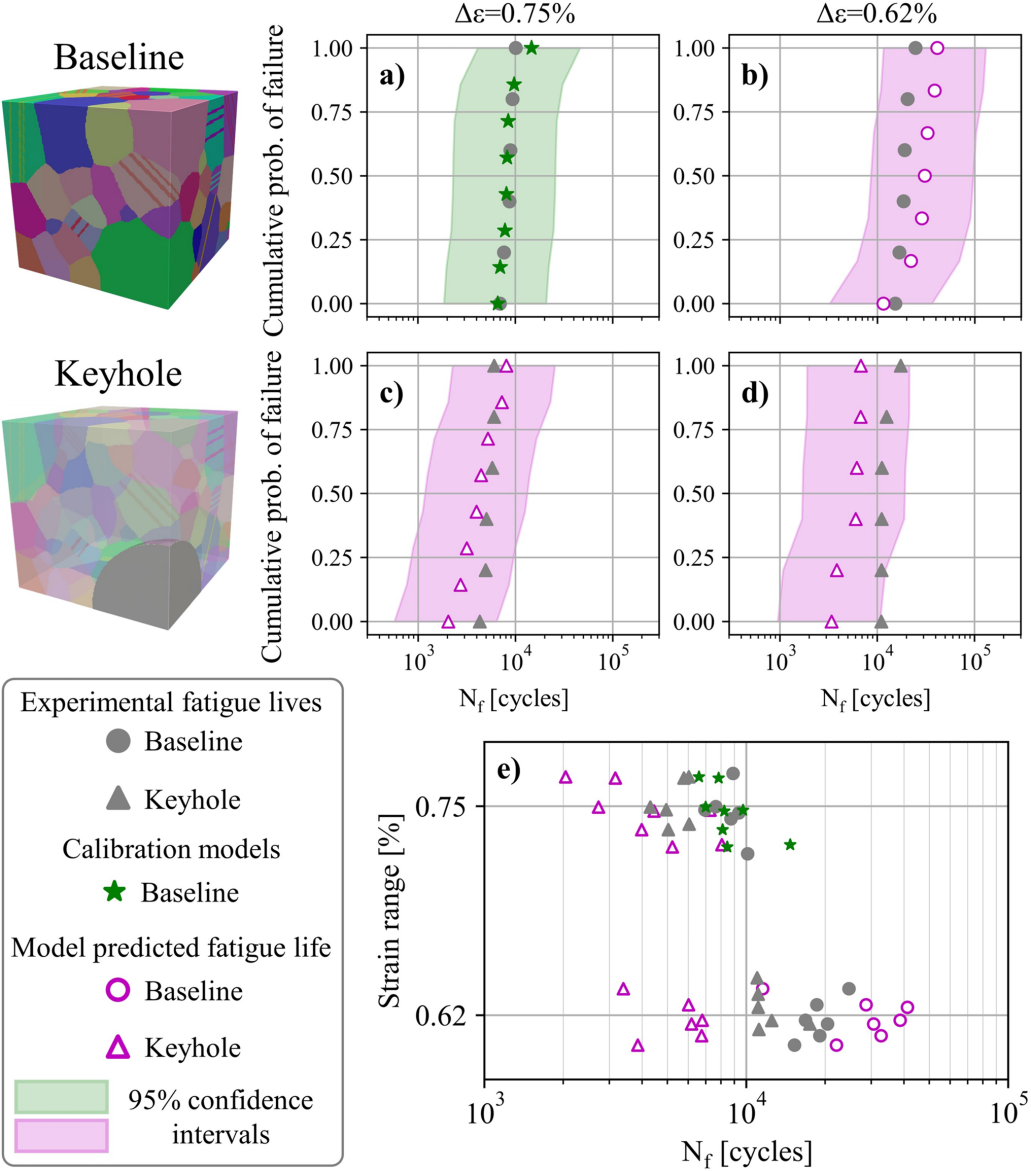
Augmenting experimental data with microstructure-sensitive models using the framework described in section “Fatigue Modeling” and depicted in Fig. 10. Statistically equivalent microstructure models without pores and seeded with keyhole pores are generated, and the former are used alongside experimental data to calibrate the critical value of a fatigue damage parameter. The calibration leverages data at 0.75% strain (shown in green) and is subsequently used to predict the fatigue lives of models seeded with pores at both applied strains (shown in magenta). (**e**) Compiled experimental and model results. Jitter is added to prevent marker overlap

Using the framework described in this section, the fatigue life of the nominal conditions and associated debit in the fatigue life of the intentionally seeded pore defects can be assessed. This is helpful to supplement the experimental test data, to provide additional statistics, as well as expand the dataset to additional testing conditions. Figure 11 depicts experimental and model fatigue life data of the baseline and keyhole porosity conditions [37, 39]. Fully dense models representative of the baseline specimens and models seeded with keyhole pores undergo cyclic loading at the same conditions as the experiments in a CPFE simulation. The baseline models and specimens cyclically loaded to 0.75% strain are then used to calibrate the critical value of the APSED. The calibrated APSED is subsequently used to predict the fatigue lives of models seeded with keyhole pores at both applied strains and the baseline models at the lower 0.62% applied strain. As a note, the model data depicted in Fig. 11a augment experimental observations, while the model fatigue lives shown in Fig. 11b–d are predictions and match reasonably well with experimental observations. All the model and experimental data are shown collectively in Fig. 11e in which jitter is added to prevent marker overlap. These fatigue modeling results will be used in the following section to provide a more robust set of design allowables.

The CPFE fatigue modeling framework described here has been applied across fatigue regimes [23, 78, 87, 88], with specific focus on both the low cycle fatigue (LCF) [39, 89] and high cycle fatigue (HCF) [83] regimes while accounting for porosity. Notably, the HCF regime may be more sensitive to porosity, given that stress concentrations induced by pores have a more pronounced effect when plasticity at the microscale is limited. While life in the HCF regime is dominated by crack initiation, the LCF regime is dominated by fatigue crack growth, and as such it is important to consider microstructurally-sensitive fatigue crack growth rates in the context of crystal plasticity, as detailed in Refs. [90–93].

## Application of the Hybrid Methodology to Fatigue Design Allowables

Creating seeded pore defect data can be beneficial in multiple scenarios for assessing the fatigue life of AM components as it provides a means to consider unintentional variation observed within nominal process conditions. Moreover, microstructure-sensitive models provide additional regression data, enhancing confidence in the computed fatigue design allowables. Three such scenarios will be discussed in the following sections, each of which begins with a fundamental consideration of the design allowable curve discussed in section “Fatigue-Limited Parts and Design Allowables”.

The design allowable curve is intended to represent the nominal process conditions to build AM materials/components. Therefore, the design allowable may not account for test bars containing off-nominal porosity and defect concentrations. As suggested in this methodology for rapid qualification, the design allowable should have margin to adequately cover scenarios where the NDE resolution techniques cannot resolve pore defects due to off-nominal process conditions. Physical testing and modeling at nominal and off-nominal conditions and at multiple stress/strain conditions can help establish a more robust set of design allowables. Accordingly, we distinguish between a design allowable curve determined using data at nominal process conditions, i.e., the nominal design allowable curve, and a design allowable modified based on experimental and/or model data with intentionally seeded pores, i.e., the augmented design allowable curve.

In the first example below, model data are used alongside experimental data at a single loading condition to identify the statistically minimum fatigue life, which is often difficult to determine through routine coupon testing due to rare events. Subsequently, model data obtained under nominal process conditions is employed to ensure robustness in the experimentally derived fatigue design allowable curve. Finally, the fatigue design allowable curve is augmented by incorporating experimental data that includes seeded porosity.

### Identifying Statistically Minimum Fatigue Life

**Fig. 12:**
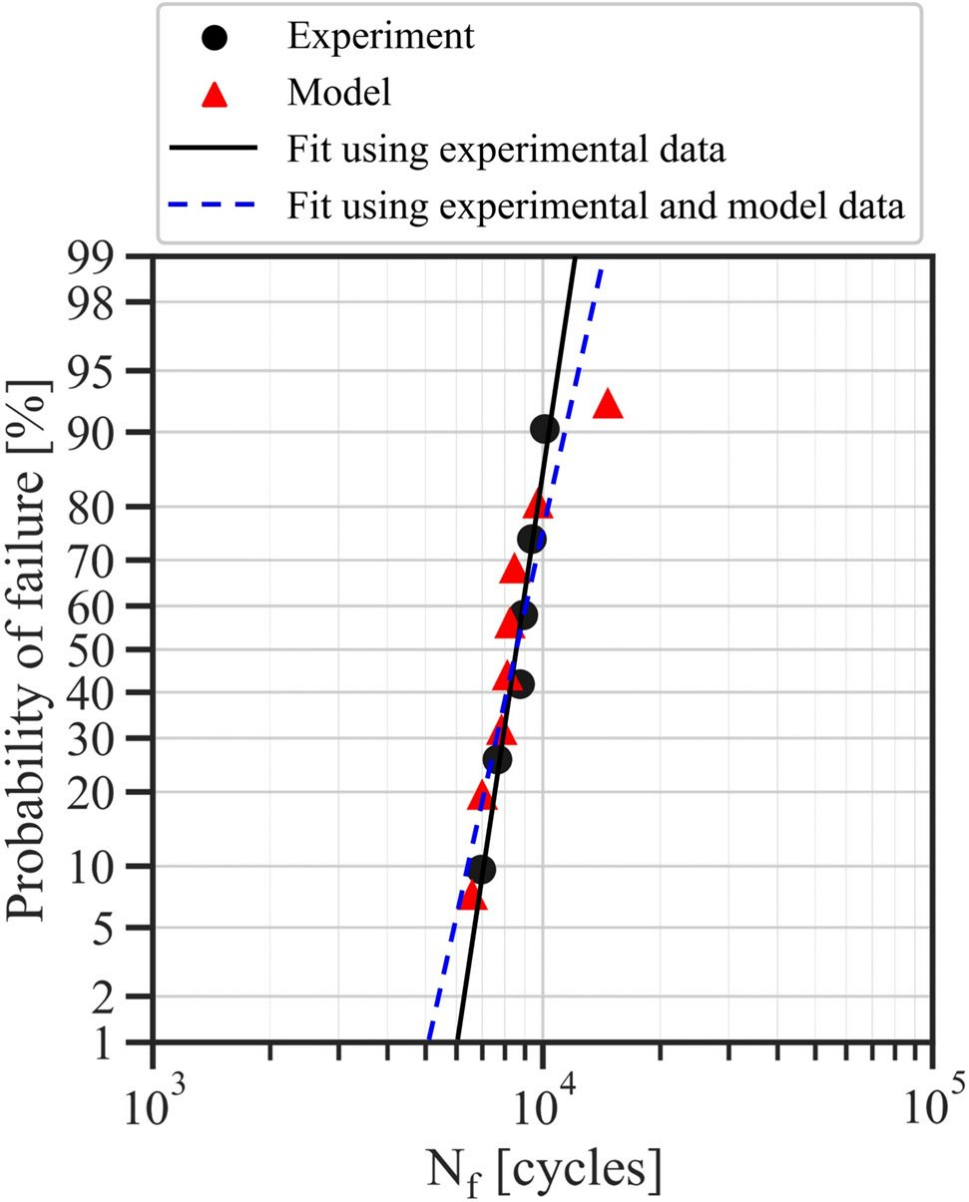
Probability of failure for additively manufactured alloy 718 specimens produced with minimal stochastic porosity alongside corresponding model data. Specimens and models were cyclically loaded to 0.75% strain at a strain ratio of *R*_*ε*_ = 0. The extrapolated log-normal fits can be used to predict the probability of failure of 1/1000 (i.e., the B0.1 life) [94]. The fit using both experimental and model data results in a higher confidence prediction

Fatigue test campaigns can be extremely costly and time consuming, and finding ways to augment existing empirical data with microstructure-based model data can help reduce overall NRE costs and effort [39]. Since the models developed have a component that is microstructurally based, additional model data for nominal conditions could be generated. Given that a fatigue design allowable heavily relies on understanding the location of a statistical minimum, as discussed in the section “Fatigue-Limited Parts and Design Allowables”, combining this “nominal” model data with empirical data for average regression and statistical minimum calculation results in a robust fatigue design allowable at nominal conditions without added experimental testing costs. Figure 12 depicts the probability of failure of AM alloy 718 specimens at a specific loading condition, in which the log-normal fit using both experimental and model data results in a higher confidence prediction of the B0.1 life [6, 37, 39, 94]. It should be noted that the data depicted in Figs. 12 and 11a are identical. The advantage of such an approach is that once the model is developed and validated, numerous statistically equivalent microstructures can be created and simulated, in order to produce a more statistically robust fatigue response and subsequent life. This is an effective means to identify tails in the fatigue life distribution and probe extreme values associated with the statistical minimum, without requiring additional testing.

### Ensuring Robustness of the Experimental Fatigue Design Allowables

**Fig. 13:**
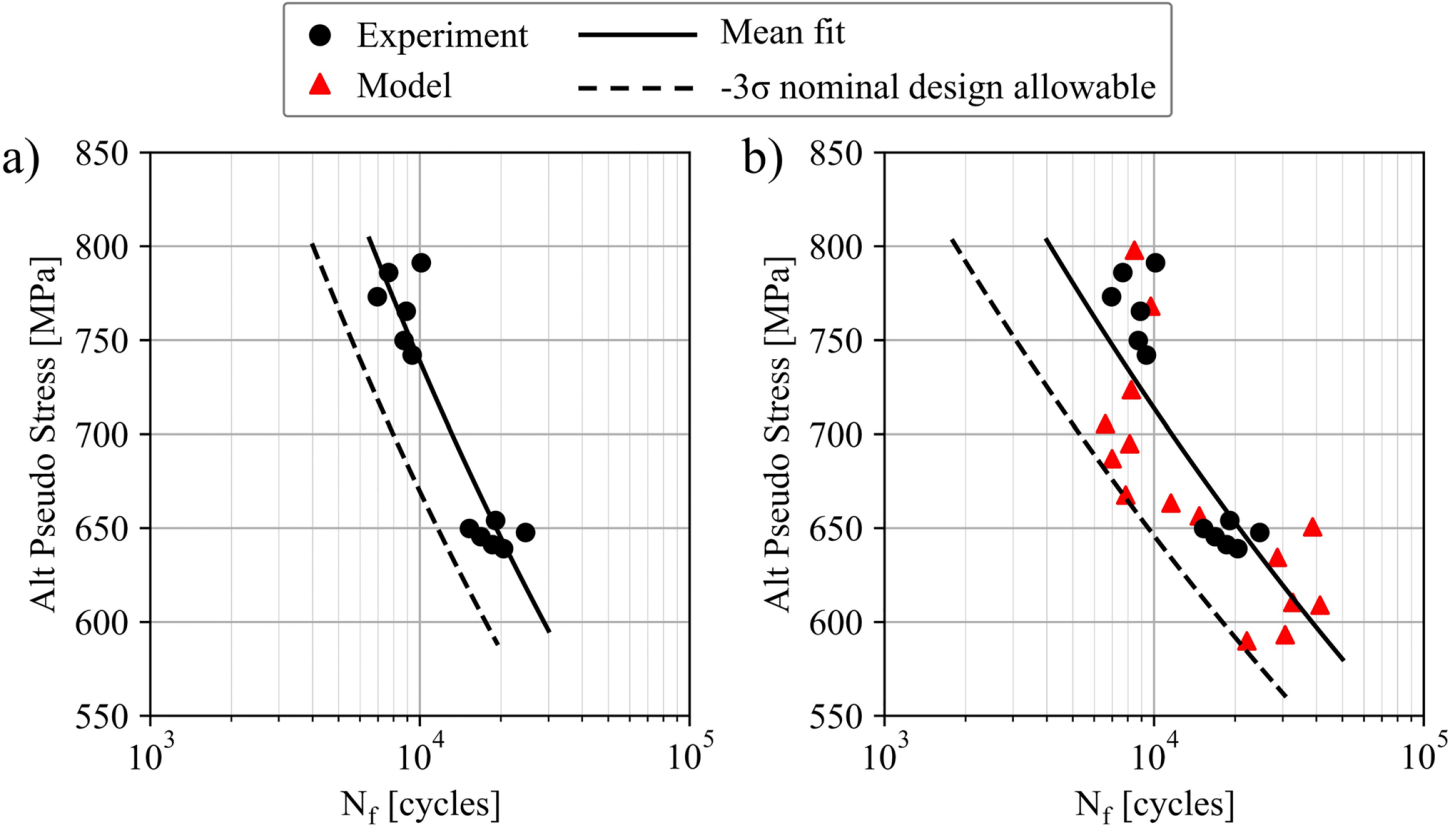
Fatigue design allowable curve for additively manufactured alloy 718 determined using **a** experimental data and **b** both experimental and model data. The model data leveraged are also depicted in Fig. 11a and b

The experimentally validated microstructure-sensitive models can be evaluated at multiple conditions, potentially extending beyond those included in the original experimental campaign, to ensure robustness of the fatigue design allowable curve. Typically, due to practical limitations in the number of experiments, the nominal fatigue design allowables only represent a set of experimental coupons representing nominal loading, microstructure, and defect structure. Once the microstructure-sensitive modeling framework is established, it is possible to further examine the effects of additional loading scenarios or microstructure variability on fatigue resistance. To ensure robustness, it is practical to compare the design allowables under additional loading configurations that are assessed by the model. Figure 13 illustrates this concept, utilizing the experimental and model data presented in Fig. 11a and 11b. In Fig. 13b, the fatigue design allowable curve is computed using both experimental and model data at nominal conditions. In another distinct scenario, the microstructure could vary from the standard condition, for instance due to departure from the target post-processing conditions, which often requires a materials review board assessment. In these cases, the microstructural features, such as grain size, twin density, the presence of crystallographic texture, etc., may deviate from the characterized values depicted in Fig. 8. In this second scenario, the microstructure-sensitive model can be used to assess deviations in the microstructure feature distributions influence on the fatigue design allowables, thereby assessing robustness in the design allowables.

### Augmenting Fatigue Design Allowables Using Off-Nominal Process Conditions

**Fig. 14:**
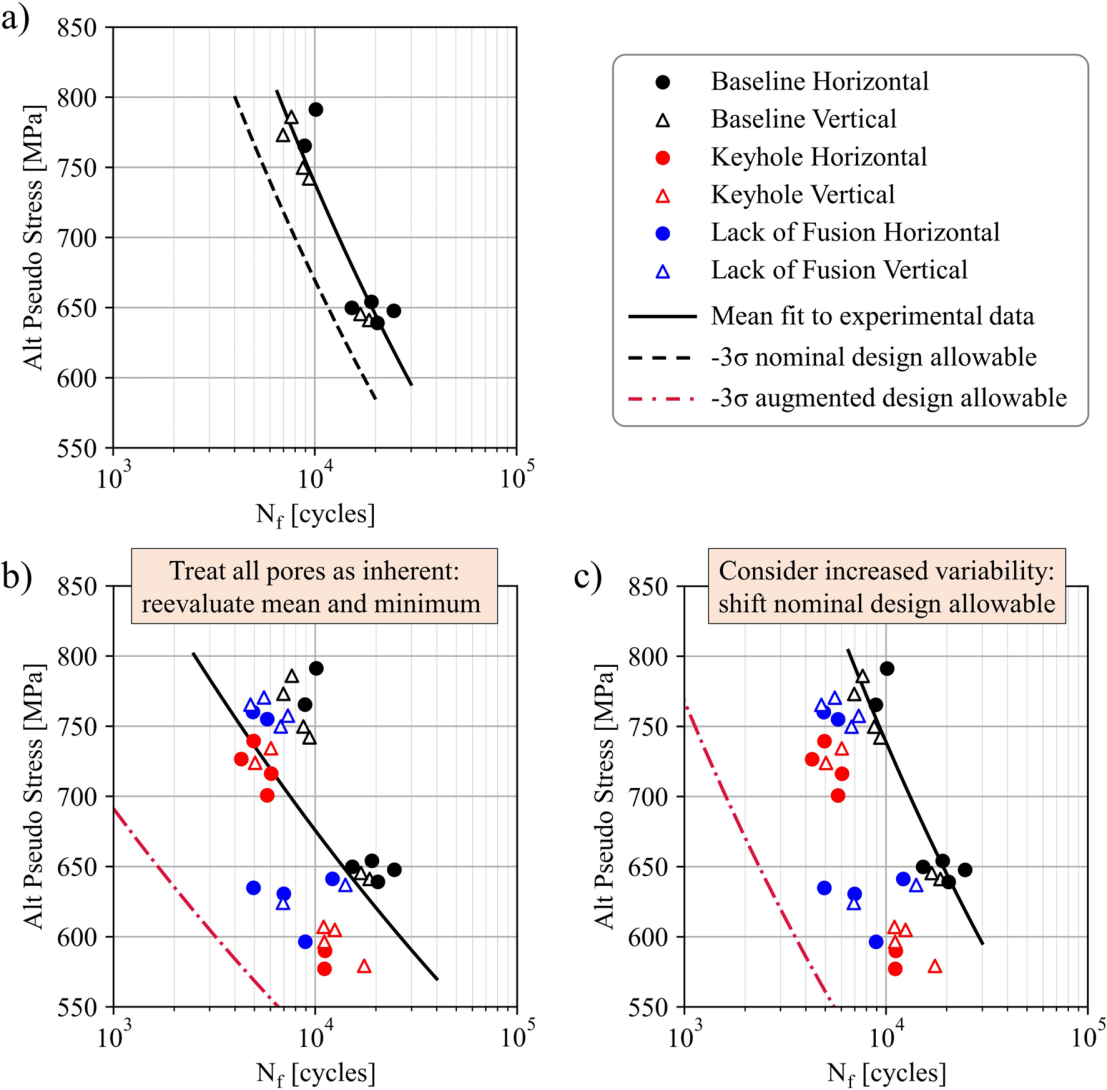
Augmenting fatigue design allowable curves using off-nominal process conditions to seed pore defects into additively manufactured alloy 718 specimens. The data in this figure come exclusively from physical experiments, and the legend indicates the specimen build orientation. **a** Experimental data of specimens produced at nominal conditions with minimal stochastic porosity. In **b** and **c**, off-nominal process conditions are employed to seed specimens with keyhole and lack of fusion pore defects. The combined data are then used to augment the fatigue design allowable curve

Testing off-nominal process conditions that represent potential AM pore defect distributions and including these distributions into an overall average regression result in a wider window of potential variation from the stochastic AM process. The combined data set of nominal and off-nominal conditions, albeit empirical and/or model based, likely results in a lower fatigue design allowable compared to a fatigue design allowable established solely from nominal test data. Figure 14 illustrates experimental enhancements to a fatigue design allowable using specimens seeded with keyhole and LOF pores representative of off-nominal process conditions [37]. Figure 14a depicts test data at nominal process conditions and the associated nominal design allowable. In Fig. 14b, data from specimens seeded with keyhole and LOF pores are considered, and the mean and minimum fits are reevaluated using all the data, resulting in a lower, augmented design allowable. On the other hand, Fig. 14c depicts an augmented design allowable in which the standard error is recomputed using all the data with respect to the mean fit of just the baseline data. The design allowables can additionally be augmented using both experimental and model data seeded with pores leveraging the microstructure-sensitive modeling framework and data depicted in Fig. 11. While testing off-nominal conditions may add to upfront NRE costs, strategic test planning and model execution can lead to downstream cost and time savings to provide a means for increasing confidence in AM material qualification.

## Application of the Hybrid Methodology for Zone-Based Life Analysis

In AM, material qualification and part certification are inherently coupled; as the part is being built, the microstructure and defects in the material will vary. For instance, contour practices and local geometric features like overhangs or internal cooling passages will influence local heat conductivity and therefore influence the presence of local defects and microstructure. Thus, standard homogeneous material qualification may not be applicable across a part. The present approach utilizes a baseline AM build process (e.g., target microstructure given the AM build parameters) and associated allowable fatigue life. For part certification, location-specific debits should be assessed on the local material fatigue allowables based on the local presence of pore defects. Moreover, this approach can be extended to include the role of bulk residual stresses [61] or variability in the microstructure [87] across the part. Hence, a zoning methodology is produced, where a part is discretized to identify the stress state in a specific region, as well as probability of defects existing in that particular region [95]. The advantage of the zoning method is that it can account for the probability of detection of such defects based on the location and part geometry.

For the structural analysis of the part, a finite element (FE) simulation can examine the role of loading applied on the part’s geometry. From the resulting spatial–temporal fields from the FE analysis, the cyclic principal stress/strain is calculated at each material point. A second sub-scale material modeling approach assesses the micromechanical response of the material via the microstructure-sensitive fatigue model (“Fatigue Modeling” section). Hence, a hierarchical approach is taken [87], where the FE analysis provides component level data, and at the critical material points, sub-scale hybrid experimental and material models are used to assess the role of local pore defects on the fatigue life. For this sub-scale material model, CPFE simulations are conducted on a series of virtual microstructures.

**Fig. 15:**
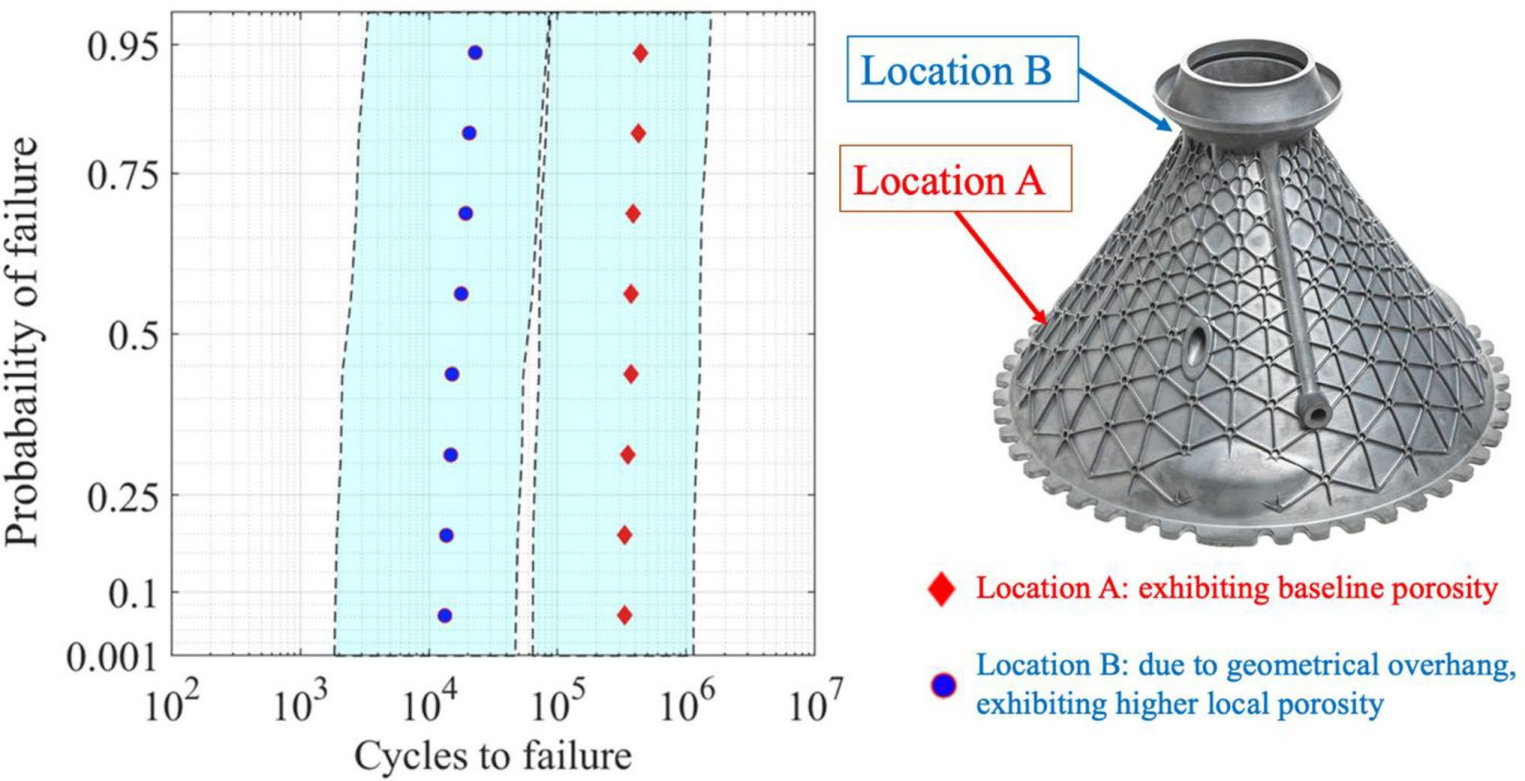
Two regions in a part are depicted: Location A exhibiting baseline low levels of porosity and Location B exhibiting higher levels of porosity due to the geometric overhang. Due to the presence of porosity and the local stress fields, the fatigue life at Location B is debited as shown, along with confidence intervals. Image of part courtesy of GE Additive

Using such an approach can enable material qualification based on a nominal set of design allowables, in which the material exhibits minimal porosity, as well as rapid assessment of the role of local porosity in specific locations across the part, based on a quantified debit in the allowable fatigue life. Figure 15 provides an illustrative example of this concept. The zoning approach enables an easy framework to account for the dependence of pore defects occurring due to different contour practices, near distinct geometrical features such as overhangs, limitations of porosity detection based on expected size and location, and a nucleating crack propagating into a dominant crack based on local stress fields and geometric correction factors.

In the augmented design allowables discussed in the section “Augmenting Fatigue Design Allowables Using Off-Nominal Process Conditions”, additional fatigue life scatter is added to the nominal set of design allowables to account for intentionally seeded pore defects that may occur from off-nominal conditions. While this additional variability may result in a more robust set of design allowables, it will reduce the fatigue life that is acceptable in design, requiring additional material (thereby increasing weight and cost and reducing potential energy efficiency) or reducing service life (adding cost). While this methodology provides rapid qualification, it could come at the expense of cost.

The benefit of the rapid material qualification guideline is a closer coupling with part certification through zone-based lifing. At specific locations within the part, advancements with process modeling tools can predict the expected pore defects [96–98], which can be integrated within the present modeling approach to provide a complete process-structure-properties-performance linkage. These path forward will be bolstered with advancements in NDE or in operando-based sensing techniques (as discussed in section “Non-Destructive Evaluation”) that will reduce the range in off-nominal process parameters required in the DOE to produce smaller intentionally seeded defects. Thus, with the close connection between material qualification and part certification in the AM process, this methodology presents a promising path forward for employing AM components into fatigue-limited applications.

## Conclusions

The variability in the additive manufacturing (AM) build process, due to the large number of process parameters (e.g., laser power and speed, powder layer thickness, hatch spacing, part orientation and location on build platform, etc.) and intricate component geometry such as overhangs and thin walls, creates porosity that poses challenges in determining reliable fatigue design allowables. Conventional material qualification approaches assume a relatively homogeneous microstructure and a uniform distribution of defects such as pores, which may not be applicable to AM components. This limitation hinders the qualification of AM materials and their adoption in fatigue-limited applications.

This work proposes a hybrid approach that combines experiments and microstructure-based simulations to facilitate rapid qualification of AM components. The hybrid approach features three aspects: (1) intentionally seeding pore defects into AM specimens based on deviations from nominal process conditions, (2) a combination of destructive (e.g., optical metallography, microstructure characterization, mechanical fatigue testing) and non-destructive (e.g., computed tomography) techniques for material characterization, and (3) microstructure-based simulations of models seeded with representative pore defects resulting from the off-nominal AM process conditions. We discuss the development of AM process parameters and fatigue design allowables and demonstrate the hybrid approach using data from Ni-base alloy 718. Supplementing experimental data obtained at nominal process conditions with specimens and models that include seeded pore defects enhances confidence in the computed fatigue life predictions. The approach is used to more rapidly assess a set of design fatigue allowables via: (1) modeling predictions to develop a larger statistical distribution to identify the statistically minimum fatigue life, (2) model predictions to examine additional loading conditions or deviations outside microstructure specifications beyond the experimental test campaign, and (3) seeded defect materials to develop a more robust set of design allowables beyond the bulk, nominal processing conditions. Moreover, the presented approach enables zone-based life analysis by considering variations in defect distribution and microstructure due to local geometric features such as overhangs or thin walls. In the zone base lifing approach, a closer connection between material qualification and part certification is established. The porosity defect distribution can be identified at specific locations near geometric features, either via characterization, process modeling, or in operando sensing within a part. Afterward, via seeded defect pore defect experimental tests and/or microstructure-based modeling, coupled with knowledge of the stress state at the specific location of the part, the fatigue life is predicted, in order to provide a location specific life distribution across the part.

## References

[1] Seifi M, Gorelik M, Waller J, Hrabe N (2017). Progress towards metal additive manufacturing standardization to support qualification and certification. JOM.

[2] Gorelik M (2017). Additive manufacturing in the context of structural integrity. Int J Fatigue.

[3] Seifi M, Salem A, Beuth J, Harrysson O (2016). Overview of materials qualification needs for metal additive manufacturing. JOM.

[4] Russell R, Wells D, Waller J, Poorganji B, Froes F, Boyer R (2019). 3-qualification and certification of metal additive manufactured hardware for aerospace applications. Additive manufacturing for the aerospace industry.

[5] Bannantine JA, Comer JJ, Handrock JL (1990). Fundamentals of metal fatigue analysis.

[6] Sangid MD (2020). Coupling in situ experiments and modeling—opportunities for data fusion, machine learning, and discovery of emergent behavior. Curr Opin Solid State Mater Sci.

[7] Lavenstein S, El-Awady JA (2019). Micro-scale fatigue mechanisms in metals: Insights gained from small-scale experiments and discrete dislocation dynamics simulations. Curr Opin Solid State Mater Sci.

[8] Zhang T, Jiang J, Britton B, Shollock B (2016). Crack nucleation using combined crystal plasticity modelling, high-resolution digital image correlation and high-resolution electron backscatter diffraction in a superalloy containing non-metallic inclusions under fatigue. Proc Math Phys Eng Sci.

[9] Salvati E, Lunt AJG, Heason CP, Baxter GJ (2020). An analysis of fatigue failure mechanisms in an additively manufactured and shot peened in 718 nickel superalloy. Mater Des.

[10] Ardi DT, Guowei L, Maharjan N, Mutiargo B (2020). Effects of post-processing route on fatigue performance of laser powder bed fusion inconel 718. Addit Manuf.

[11] Balachandramurthi AR, Moverare J, Dixit N, Pederson R (2018). Influence of defects and as-built surface roughness on fatigue properties of additively manufactured alloy 718. Mater Sci Eng A.

[12] Sanaei N, Fatemi A (2021). Defect-based fatigue life prediction of l-pbf additive manufactured metals. Eng Fract Mech.

[13] Sanaei N, Fatemi A (2021). Defects in additive manufactured metals and their effect on fatigue performance: a state-of-the-art review. Prog Mater Sci.

[14] Sanaei N, Fatemi A, Phan N (2019). Defect characteristics and analysis of their variability in metal l-pbf additive manufacturing. Mater Des.

[15] Tillmann W, Schaak C, Nellesen J, Schaper M (2017). Hot isostatic pressing of in718 components manufactured by selective laser melting. Addit Manuf.

[16] Poulin JR, Kreitcberg A, Brailovski V (2021). Effect of hot isostatic pressing of laser powder bed fused inconel 625 with purposely induced defects on the residual porosity and fatigue crack propagation behavior. Addit Manuf.

[17] du Plessis A, Macdonald E (2020). Hot isostatic pressing in metal additive manufacturing: X-ray tomography reveals details of pore closure. Addit Manuf.

[18] Thapliyal S, Mishra RS, Kadkhodapour J, Schmauder S, Sajadi F (2023). Chapter eight—linking materials systems approach to alloy design and part qualification for laser powder bed fusion additive manufacturing. Quality analysis of additively manufactured metals.

[19] Olson GB (1997). Computational design of hierarchically structured materials. Science.

[20] Peralta AD, Enright M, Megahed M, Gong J (2016). Towards rapid qualification of powder-bed laser additively manufactured parts. Integr Mater Manuf Innov.

[21] Gong J, Deutchman HZ, Peralta A, Snyder D (2016). Integrated thermal process optimization of alloy 718plus® for additive manufacturing. Superalloys.

[22] Mindt HW, Desmaison O, Megahed M, Peralta A (2018). Modeling of powder bed manufacturing defects. J Mater Eng Perform.

[23] Bandyopadhyay R, Prithivirajan V, Peralta AD, Sangid MD (2020). Microstructure-sensitive critical plastic strain energy density criterion for fatigue life prediction across various loading regimes. Proc Math Phys Eng Sci.

[24] Megahed M, Mindt H-W, Willems J, Dionne P (2019). LPBF
right the first time—the right mix between modeling and experiments. Integr Mater Manuf Innov.

[25] Pires P-A, Desmaison O, Megahed M (2018). Icme manufacturability assessment in powder bed fusion additive manufacturing. JOM.

[26] Zielinski J, Mindt H-W, Düchting J, Schleifenbaum JH (2017). Numerical and experimental study of ti6al4v components manufactured using powder bed fusion additive manufacturing. JOM.

[27] Hensley C, Sisco K, Beauchamp S, Godfrey A (2021). Qualification pathways for additively manufactured components for nuclear applications. J Nucl Mater.

[28] Jalalahmadi B, Liu J, Liu Z, Vechart A (2021). An integrated computational materials engineering predictive platform for fatigue prediction and qualification of metallic parts built with additive manufacturing. J Tribol.

[29] Standard practice for strain-controlled fatigue testing. ASTM E606. https://www.astm.org/standards/e606

[30] Standard practice for conducting force controlled constant amplitude axial fatigue tests of metallic materials. ASTM E466. https://www.astm.org/e0466-21.html

[31] Fintová S, Kuběna I, Trško L, Horník V (2020). Fatigue behavior of aw7075 aluminum alloy in ultra-high cycle fatigue region. Mater Sci Eng A.

[32] Szczepanski CJ, Jha SK, Larsen JM, Jones JW (2008). Microstructural influences on very-high-cycle fatigue-crack initiation in ti-6246. Metall Mater Trans A.

[33] Ravi Chandran KS, Jha SK (2005). Duality of the s–n fatigue curve caused by competing failure modes in a titanium alloy and the role of poisson defect statistics. Acta Mater.

[34] Lee Y-L, Taylor D, Lee Y-L, Pan JWO, Hathaway RB, Barkey ME (2005). 4-Stress-based fatigue analysis and design. Fatigue testing and analysis.

[35] Altman DG, Bland JM (2005). Standard deviations and standard errors. BMJ.

[36] Solberg K, Wan D, Berto F (2020). Fatigue assessment of as-built and heat-treated inconel 718 specimens produced by additive manufacturing including notch effects. Fatigue Fract Eng Mater Struct.

[37] Stopka KS, Desrosiers A, Nicodemus T, Krutz N (2023). Intentionally seeding pores in additively manufactured alloy 718: process parameters, microstructure, defects, and fatigue. Addit Manuf.

[38] Scime L, Beuth J (2019). Melt pool geometry and morphology variability for the inconel 718 alloy in a laser powder bed fusion additive manufacturing process. Addit Manuf.

[39] Stopka KS, Sangid MD (2023). Modeling fatigue behavior of additively manufactured alloys with an emphasis on pore defect morphology. J Mech Phys Solids.

[40] Cunningham R, Nicolas A, Madsen J, Fodran E (2017). Analyzing the effects of powder and post-processing on porosity and properties of electron beam melted Ti–6AL–4V. Mater Res Lett.

[41] Quintana M, Ji Y, Collins P (2022). A perspective of the needs and opportunities for coupling materials science and nondestructive evaluation for metals-based additive manufacturing. Mater Eval.

[42] Astm wk75329 (2022) New practice for nondestructive testing (NDT), Part quality, and Acceptability levels of additively manufactured laser based powder bed fusion aerospace components, West Conshohocken. Work in Progress. https://www.astm.org/workitem-wk75329

[43] Maher M (2016) In ARPA-E METALS annual meeting, Detroit, MI. Open manufacturing overview, vol. 23. Defense Advanced Research Projects Agency

[44] Mankins JC (2009). Technology readiness and risk assessments: a new approach. Acta Astronaut.

[45] Mankins JC (1995) Technology readiness levels: a white paper

[46] King WE, Barth HD, Castillo VM, Gallegos GF (2014). Observation of keyhole-mode laser melting in laser powder-bed fusion additive manufacturing. J Mater Process Technol.

[47] Khorasani M, Ghasemi A, Leary M, Sharabian E (2022). The effect of absorption ratio on meltpool features in laser-based powder bed fusion of in718. Opt Laser Technol.

[48] Hann DB, Iammi J, Folkes J (2011). A simple methodology for predicting laser-weld properties from material and laser parameters. J Phys D: Appl Phys.

[49] Sangid MD, Book TA, Naragani D, Rotella J (2018). Role of heat treatment and build orientation in the microstructure sensitive deformation characteristics of in718 produced via slm additive manufacturing. Addit Manuf.

[50] Bachmann F, Hielscher R, Schaeben H (2010) In Solid state phenomena. Texture analysis with mtex–free and open source software toolbox, vol 160. Trans Tech Publ., pp 63–68

[51] Azinpour E, Darabi R, Cesar de Sa J, Santos A (2020). Fracture analysis in directed energy deposition (ded) manufactured 316l stainless steel using a phase-field approach. Finite Elem Anal Des.

[52] Cui Y, Gao YF, Chew HB (2020). Two-scale porosity effects on cohesive crack growth in a ductile media. Int J Solids Struct.

[53] Karpenko O, Oterkus S, Oterkus E (2021). Peridynamic investigation of the effect of porosity on fatigue nucleation for additively manufactured titanium alloy ti6al4v. Theor Appl Fract Mech.

[54] Bercelli L, Moyne S, Dhondt M, Doudard C (2021). A probabilistic approach for high cycle fatigue of wire and arc additive manufactured parts taking into account process-induced pores. Addit Manuf.

[55] Haridas RS, Thapliyal S, Agrawal P, Mishra RS (2020). Defect-based probabilistic fatigue life estimation model for an additively manufactured aluminum alloy. Mater Sci Eng A.

[56] Kapoor R, Rao VSH, Mishra RS, Baumann JA (2011). Probabilistic fatigue life prediction model for alloys with defects: applied to a206. Acta Mater.

[57] Asaro RJ (1983). Crystal plasticity. J Appl Mech.

[58] Quey R, Dawson PR, Barbe F (2011). Large-scale 3d random polycrystals for the finite element method: generation, meshing and remeshing. Comput Methods Appl Mech Eng.

[59] Quey R, Renversade L (2018). Optimal polyhedral description of 3d polycrystals: method and application to statistical and synchrotron X-ray diffraction data. Comput Methods Appl Mech Eng.

[60] Groeber MA, Jackson MA (2014). Dream. 3d: a digital representation environment for the analysis of microstructure in 3d. Integr Mater Manuf Innov.

[61] Kapoor K, Sangid MD (2018). Initializing type-2 residual stresses in crystal plasticity finite element simulations utilizing high-energy diffraction microscopy data. Mater Sci Eng A.

[62] Yaghoobi M, Stopka KS, Lakshmanan A, Sundararaghavan V (2021). Prisms-fatigue computational framework for fatigue analysis in polycrystalline metals and alloys. NPJ Comput Mater.

[63] Yaghoobi M, Ganesan S, Sundar S, Lakshmanan A (2019). Prisms-plasticity: An open-source crystal plasticity finite element software. Comput Mater Sci.

[64] Roters F, Diehl M, Shanthraj P, Eisenlohr P (2019). Damask – the düsseldorf advanced material simulation kit for modeling multi-physics crystal plasticity, thermal, and damage phenomena from the single crystal up to the component scale. Comput Mater Sci.

[65] Quey R, Kasemer M (2022). The neper/fepx project: free/open-source polycrystal generation, deformation simulation, and post-processing. IOP Conf Ser Mater Sci Eng.

[66] Patra A, Chaudhary S, Pai N, Ramgopal T (2023). Ρ-cp: Open source dislocation density based crystal plasticity framework for simulating temperature- and strain rate-dependent deformation. Comput Mater Sci.

[67] Cowles B, Backman D, Dutton R (2012). Verification and validation of icme methods and models for aerospace applications. Integr Mater Manuf Innov.

[68] Liu X, Furrer D, Kosters J, Holmes J (2018) Vision 2040: a roadmap for integrated, multiscale modeling and simulation of materials and systems. NASA, Washington, DC. https://ntrs.nasa.gov/citations/20180002010

[69] Li DY, Szpunar JA (1992). Determination of single crystals' elastic constants from the measurement of ultrasonic velocity in the polycrystalline material. Acta Metall et Mater.

[70] Purushottamrajpurohit RRP, Richeton T, Berbenni S, Germain L (2021). Estimating single-crystal elastic constants of polycrystalline β metastable titanium alloy: a bayesian inference analysis based on high energy x-ray diffraction and micromechanical modeling. Acta Mater.

[71] Dryburgh P, Li W, Pieris D, Fuentes-Domínguez R (2022). Measurement of the single crystal elasticity matrix of polycrystalline materials. Acta Mater.

[72] Shenoy M, Tjiptowidjojo Y, McDowell D (2008). Microstructure-sensitive modeling of polycrystalline in 100. Int J Plast.

[73] Bandyopadhyay R, Prithivirajan V, Sangid MD (2019). Uncertainty quantification in the mechanical response of crystal plasticity simulations. JOM.

[74] Hochhalter J, Bomarito G, Yeratapally S, Leser P et al (2020) Non-deterministic calibration of crystal plasticity model parameters. In: Ghosh S, Woodward C, Przybyla C (eds) Integrated computational materials engineering (ICME): advancing computational and experimental methods. Springer, Cham, pp 165–198. 10.1007/978-3-030-40562-5_6

[75] Pagan DC, Shade PA, Barton NR, Park J-S (2017). Modeling slip system strength evolution in ti-7al informed by in-situ grain stress measurements. Acta Mater.

[76] Bandyopadhyay R, Gustafson SE, Kapoor K, Naragani D (2021). Comparative assessment of backstress models using high-energy x-ray diffraction microscopy experiments and crystal plasticity finite element simulations. Int J Plast.

[77] Hennessey C, Castelluccio GM, McDowell DL (2017). Sensitivity of polycrystal plasticity to slip system kinematic hardening laws for al 7075–t6. Mater Sci Eng A.

[78] Bandyopadhyay R, Sangid MD (2021). A probabilistic fatigue framework to enable location-specific lifing for critical thermo-mechanical engineering applications. Integr Mater Manuf Innov.

[79] Shenoy M, Zhang J, McDowell DL (2007). Estimating fatigue sensitivity to polycrystalline ni-base superalloy microstructures using a computational approach. Fatigue Fract Eng Mater Struct.

[80] Fatemi A, Socie DF (1988). A critical plane approach to multiaxial fatigue damage including out-of-phase loading. Fatigue Fract Eng Mater Struct.

[81] Chen B, Jiang J, Dunne FPE (2018). Is stored energy density the primary meso-scale mechanistic driver for fatigue crack nucleation?. Int J Plast.

[82] Anahid M, Samal MK, Ghosh S (2011). Dwell fatigue crack nucleation model based on crystal plasticity finite element simulations of polycrystalline titanium alloys. J Mech Phys Solids.

[83] Prithivirajan V, Sangid MD (2020). Examining metrics for fatigue life predictions of additively manufactured in718 via crystal plasticity modeling including the role of simulation volume and microstructural constraints. Mater Sci Eng A.

[84] Prithivirajan V, Ravi P, Naragani D, Sangid MD (2021). Direct comparison of microstructure-sensitive fatigue crack initiation via crystal plasticity simulations and in situ high-energy x-ray experiments. Mater Des.

[85] Behnam A, Truster TJ, Tipireddy R, Messner MC (2022). Uncertainty quantification framework for predicting material response with large number of parameters: Application to creep prediction in ferritic-martensitic steels using combined crystal plasticity and grain boundary models. Integr Mater Manuf Innov.

[86] Venkatraman A, McDowell DL, Kalidindi SR (2022). Bayesian analysis of parametric uncertainties and model form probabilities for two different crystal plasticity models of lamellar grains in α+β titanium alloys. Int J Plast.

[87] Gopalakrishnan S, Bandyopadhyay R, Sangid MD (2022). A framework to enable microstructure-sensitive location-specific fatigue life analysis of components and connectivity to the product lifecycle. Int J Fatigue.

[88] Krishnamoorthi S, Bandyopadhyay R, Sangid MD (2023). A microstructure-based fatigue model for additively manufactured Ti–6AL–4V, including the role of prior β boundaries. Int J Plast.

[89] Prithivirajan V, Sangid MD (2018). The role of defects and critical pore size analysis in the fatigue response of additively manufactured in718 via crystal plasticity. Mater Des.

[90] Musinski WD, McDowell DL (2016). Simulating the effect of grain boundaries on microstructurally small fatigue crack growth from a focused ion beam notch through a three-dimensional array of grains. Acta Mater.

[91] Wilson D, Wan W, Dunne FPE (2019). Microstructurally-sensitive fatigue crack growth in hcp, bcc and fcc polycrystals. J Mech Phys Solids.

[92] Wilson D, Zheng Z, Dunne FPE (2018). A microstructure-sensitive driving force for crack growth. J Mech Phys Solids.

[93] Wilson D, Dunne FPE (2019). A mechanistic modelling methodology for microstructure-sensitive fatigue crack growth. J Mech Phys Solids.

[94] Larsen JM, Jha SK, Szczepanski CJ, Caton MJ (2013). Reducing uncertainty in fatigue life limits of turbine engine alloys. Int J Fatigue.

[95] Enright MP, McClung RC, Liang W, Lee Y-D et al (2012) In ASME Turbo Expo 2012: turbine technical conference and exposition. A tool for probabilistic damage tolerance of hole features in turbine engine rotors, vol 7: structures and dynamics, Parts A and B, pp 447–458

[96] Vastola G, Pei QX, Zhang YW (2018). Predictive model for porosity in powder-bed fusion additive manufacturing at high beam energy regime. Addit Manuf.

[97] Du Y, Mukherjee T, DebRoy T (2021). Physics-informed machine learning and mechanistic modeling of additive manufacturing to reduce defects. Appl Mater Today.

[98] Wei HL, Mukherjee T, Zhang W, Zuback JS (2021). Mechanistic models for additive manufacturing of metallic components. Prog Mater Sci.

